# Involvement of Lgl and Mahjong/VprBP in Cell Competition

**DOI:** 10.1371/journal.pbio.1000422

**Published:** 2010-07-13

**Authors:** Yoichiro Tamori, Carl Uli Bialucha, Ai-Guo Tian, Mihoko Kajita, Yi-Chun Huang, Mark Norman, Nicholas Harrison, John Poulton, Kenzo Ivanovitch, Lena Disch, Tao Liu, Wu-Min Deng, Yasuyuki Fujita

**Affiliations:** 1 Department of Biological Science, Florida State University, Tallahassee, Florida, United States of America; 2 MRC Laboratory for Molecular Cell Biology and Cell Biology Unit, University College London, London, United Kingdom; 3 Department of Cell and Developmental Biology, University College London, London, United Kingdom; 4 Division of Molecular Oncology, Institute for Genetic Medicine, Hokkaido University, Hokkaido, Japan; Albert Einstein College of Medicine, United States of America

## Abstract

During the initial stages of carcinogenesis, transformation events occur in a single cell within an epithelial monolayer. However, it remains unknown what happens at the interface between normal and transformed epithelial cells during this process. In *Drosophila*, it has been recently shown that normal and transformed cells compete with each other for survival in an epithelial tissue; however the molecular mechanisms whereby “loser cells” undergo apoptosis are not clearly understood. Lgl (lethal giant larvae) is a tumor suppressor protein and plays a crucial role in oncogenesis in flies and mammals. Here we have examined the involvement of Lgl in cell competition and shown that a novel Lgl-binding protein is involved in Lgl-mediated cell competition. Using biochemical immunoprecipitation methods, we first identified Mahjong as a novel binding partner of Lgl in both flies and mammals. In *Drosophila*, Mahjong is an essential gene, but zygotic *mahjong* mutants (*mahj* ^−/−^) do not have obvious patterning defects during embryonic or larval development. However, *mahj* ^−/−^ cells undergo apoptosis when surrounded by wild-type cells in the wing disc epithelium. Importantly, comparable phenomena also occur in Mahjong-knockdown mammalian cells; Mahjong-knockdown Madin-Darby canine kidney epithelial cells undergo apoptosis, only when surrounded by non-transformed cells. Similarly, apoptosis of *lgl* ^−/−^ cells is induced when they are surrounded by wild-type cells in *Drosophila* wing discs. Phosphorylation of the c-Jun N-terminal kinase (JNK) is increased in *mahj* ^−/−^ or *lgl* ^−/−^ mutant cells, and expression of Puckered (Puc), an inhibitor of the JNK pathway, suppresses apoptosis of these mutant cells surrounded by wild-type cells, suggesting that the JNK pathway is involved in *mahj*- or *lgl*-mediated cell competition. Finally, we have shown that overexpression of Mahj in *lgl* ^−/−^ cells strongly suppresses JNK activation and blocks apoptosis of *lgl* ^−/−^ cells in the wild-type wing disc epithelium. These data indicate that Mahjong interacts with Lgl biochemically and genetically and that Mahjong and Lgl function in the same pathway to regulate cellular competitiveness. As far as we are aware, this is the first report that cell competition can occur in a mammalian cell culture system.

## Abstract 2

Mahjong is a novel Lethal giant larvae-binding protein that plays a vital role in cell competition in both flies and mammals.

## Author Summary

Cell transformation arises from the activation of oncoproteins and/or inactivation of tumor suppressor proteins. During the initial stage of carcinogenesis, transformation occurs in a single epithelial cell that grows within an epithelial monolayer. However, it remains unclear what happens at the interface between normal and transformed epithelial cells during this process. In *Drosophila*, it has been shown that normal and transformed cells often compete with each other for survival in an epithelial tissue, in a process called “cell competition.” Lethal giant larvae (Lgl) is a tumor suppressor protein in flies and mammals. Using biochemical methods, we identified Mahjong as a novel binding partner of Lgl in flies and mammals. Furthermore, we have demonstrated that Mahjong is involved in cell competition in both flies and mammals. In particular, we found that canine kidney epithelial cells depleted for Mahjong undergo apoptosis, but only when surrounded by non-transformed cells. This represents the first evidence that cell competition can occur in a mammalian cell culture system. Although it is not clear at present what molecules/signaling pathways are regulated by Lgl/Mahjong during cell competition, future studies might reveal important pathway components that could be targeted therapeutically to prevent tumor cells from “winning” in their race against normal tissue cells.

## Introduction

Cell transformation arises from the activation of oncoproteins and/or inactivation of tumor suppressor proteins [1]. During the initial stage of carcinogenesis, transformation occurs in a single epithelial cell that grows within an epithelial monolayer [2],[3]. However, it remains unclear what happens at the interface between normal and transformed epithelial cells during this process. In *Drosophila*, it has been recently reported that normal and transformed cells compete with each other for survival in a monolayer of epithelial cells, in a process called “cell competition” [4],[5]. For example, when dMyc-overexpressing cells are surrounded by wild-type cells in an epithelial cell sheet, the surrounding wild-type cells die by apoptosis, and the dMyc-overexpressing cells proliferate to fill the vacated space [6],[7]. In contrast, when *scribble* mutant cells are surrounded by wild-type cells, *scribble* mutant cells undergo apoptosis and are eliminated from the wild-type epithelial monolayer [8]. In most of the previous reports of cell competition, slowly proliferating cells undergo apoptosis when they are surrounded by fast proliferating cells. However, activation of cyclin D/Cdk4 or the Insulin/Insulin-like growth factor receptor pathway does not cause cell competition [6], suggesting that a difference in cell growth speed alone does not always trigger cell competition. Molecular mechanisms whereby “loser cells” undergo apoptosis therefore remain largely unknown. Furthermore, it is not known whether comparable phenomena are also observed in mammalian cells.

Lethal giant larvae (Lgl) was originally identified as a tumor suppressor protein in *Drosophila* [9]. In *Drosophila* imaginal discs, mutation of *lgl* causes loss of apicobasal polarity and uncontrolled proliferation [9],[10],[11], leading to neoplastic tumor formation. In mice, knockout of Lgl1, a mammalian homologue of Lgl, was reported to result in severe brain dysplasia characterized by hyperproliferation and loss of cell polarity of neuroepithelial cells [12]. These data indicate that Lgl plays an important role in cell polarity and cell proliferation. Involvement of Lgl in cell competition was also recently reported [13]; in *Drosophila* eye discs bearing *lgl* mutant clones (*lgl* ^−^) in a mosaic manner, some *lgl* ^−^ and wild-type cells at the clone boundary undergo apoptosis and are excluded from the epithelia.

Lgl has the characteristics of a molecular scaffold, with WD protein-protein interaction motifs and no known enzymatic activity. Its functional roles are therefore likely to be mediated by protein-protein interactions. Lgl binds to atypical protein kinase C (aPKC) and Par6, and this interaction has been shown to regulate the function of Lgl in cell polarity [14],[15],[16]. However, Lgl-binding partners that are involved in Lgl-mediated cell competition have not yet been identified. In this study, we have identified viral protein R-binding protein (VprBP)/Mahjong as a novel Lgl-binding protein and have demonstrated that Lgl and VprBP/Mahjong are involved in cell competition.

## Results/Discussion

**Figure 1:**
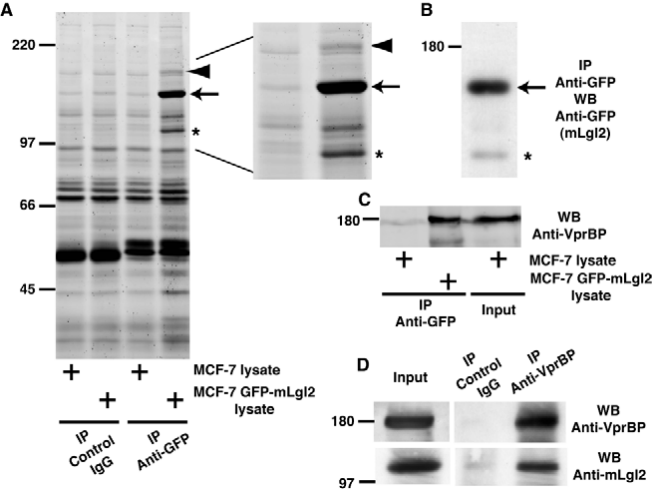
VprBP is a novel mLgl2-binding protein. (A) Identification of VprBP as an mLgl2-binding protein by immunoprecipitation. Immunoprecipitation was performed with lysates from parental or GFP-mLgl2-expressing MCF-7 epithelial cells with control mouse IgG or anti-GFP antibody, followed by SDS-PAGE and SYPRO Ruby protein staining. A magnified image of the last two lanes is shown in the right panel. (B) A degradation product of mLgl2 in the GFP-immunoprecipitate. The GFP-immunoprecipitate (the same used for the last lane in A) was examined by Western blotting with anti-GFP antibody. (A and B) Arrowhead, arrow, and asterisk indicate the positions of VprBP, GFP-mLgl2, and a degradation product of GFP-mLgl2, respectively. (C) Confirmation that VprBP is an mLgl2-binding protein. Immunoprecipitation was performed with anti-GFP antibody as described above, followed by Western blotting with anti-VprBP antibody. (D) Coimmunoprecipitation between endogenous mLgl2 and VprBP proteins. Immunoprecipitation was performed with lysates from Madin-Darby canine kidney (MDCK) epithelial cells with control rabbit IgG or anti-VprBP antibody, followed by Western blotting with anti-VprBP or anti-mLgl2 antibody.

To identify novel interacting proteins of mammalian Lgl (mLgl), we performed immunoprecipitation using MCF-7 epithelial cells stably expressing GFP-tagged mLgl2. We found two proteins (arrowhead and asterisk in Figure 1A) that were coimmunoprecipitated with GFP-mLgl2 (arrow in Figure 1A). Western blotting with anti-GFP antibody suggested that one of the two proteins was a cleavage product of GFP-mLgl2 (Figure 1B; asterisk). Mass spectrometric analysis identified the other protein, which was 180 kD in size (Figure 1A; arrowhead), as VprBP. VprBP was originally identified as interacting with human immunodeficiency virus type 1 (HIV-1) viral protein R (Vpr) [17]. VprBP interacts with the Cullin4-DDB1 ubiquitin ligase complex and regulates a Vpr-mediated induction of G2 arrest [18],[19],[20],[21], but the endogenous function of VprBP is not clearly understood. By Western blotting with anti-VprBP antibody, we confirmed the coimmunoprecipitation of VprBP with GFP-mLgl2 (Figure 1C). We also detected an interaction between endogenous VprBP and mLgl2 proteins in Madin-Darby canine kidney (MDCK) epithelial cells by immunoprecipitation with anti-VprBP antibody (Figure 1D). VprBP contains a centrally located Lis1 homology motif (LisH) as well as WD40-like domains in the C-terminal half (Figure S1A). The LisH motif is proposed to play a role in protein dimerization or microtubule dynamics [22], whereas WD40-like domains are involved in mediating protein-protein interactions [23]. Using truncated mutants, we showed that the C-terminus of mLgl2 (amino acids 544–1027) and the C-terminus of VprBP (amino acids 614–1058) are responsible for the interaction (Figure S1B and S1C).

**Figure 2:**
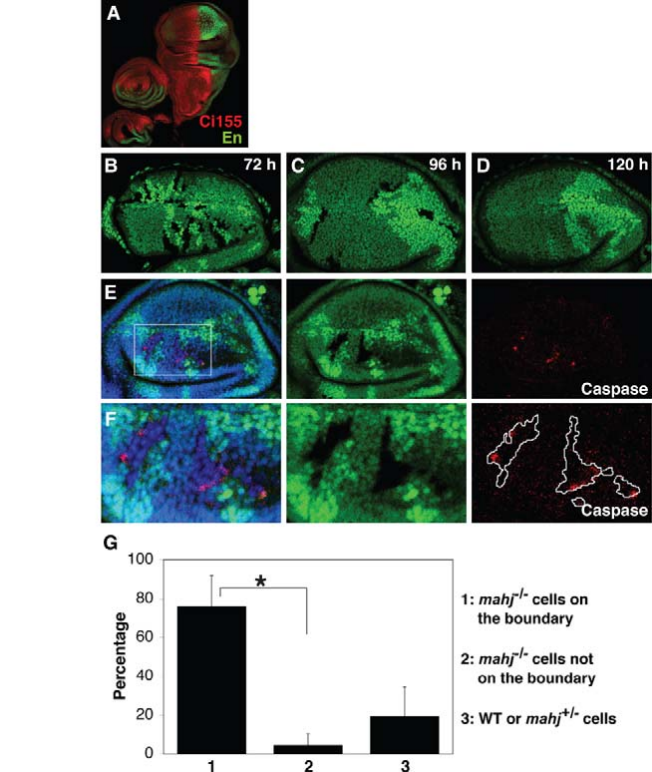
Loss of Mahj function induces cell competition in *Drosophila* wing disc epithelium. (A) Wing and leg discs of homozygous *mahj^1^* mutant third-instar *Drosophila* larvae stained with anti-Ci155 (red) and anti-Engrailed (green) antibodies. (B–F) Wing discs with *mahj* ^−/−^ (lacking GFP), wild-type (expressing GFP strongly), and *mahj^+/^* ^−^ clones (expressing GFP moderately) at 72 h (B), 96 h (C, E, and F), and 120 h (D) after clone induction. (E) A Z-stack projection of confocal images of a wing disc. Basally extruded apoptotic cells were excluded from the analysis. (F) Magnified images of (E). (E and F) Apoptotic cells were labeled with anti-cleaved Caspase-3 antibody (red). Nuclei were stained with DAPI (blue). (G) Quantification of apoptotic cells in the *mahj^1^* mosaic wing discs showing non-cell-autonomous apoptosis in *mahj* ^−/−^ cells. The results represent means±SD (*n* = 34 discs, *p<0.001).

The *Drosophila melanogaster* genome encodes a protein (CG10080) with high amino acid sequence identity (38%) with human VprBP. The CG10080 protein also contains LisH and WD40-like domains. We have named this protein Mahjong, the name of a table game in which winners and losers are determined through strong competition. First, we showed that Mahjong interacts with Lgl when the two proteins are coexpressed in *Drosophila* S2R+ cells (Figure S1D). Next, to isolate mutant alleles of *mahjong* (*mahj*), we induced imprecise excision of a P-element that was inserted in the region adjacent to the *Drosophila mahj* coding sequence (Figure S2A). By examining the genomic sequence in these excision mutants, we confirmed that one mutant had a deletion in the *Drosophila mahj*-coding region. The mutant lost exons 10 and 11, part of exon 9, and the 3′-UTR of the *mahj* transcript (Figure S2A). The homozygous mutant animals developed more slowly than wild-type flies and died at a late pupal stage (Figure S2B). The mutation failed to complement *Df(2R)XE-2900*, a chromosomal deficiency in which the entire coding region of *mahj* is deleted, and larvae trans-heterozygous for the mutation and *Df(2R)XE-2900* exhibited the same retarded development phenotype as did the homozygous excision mutant. Expression of exogenous Mahj protein alleviated both the growth defect and lethality (Figure S2C). These results indicate that the phenotype of the *mahj* excision mutant is indeed caused by a loss-of-function of *mahj*. The homozygous mutant larvae did not, however, have any detectable morphological defects (unpublished data). In their imaginal discs, compartmentalization pattern was properly maintained as observed with the antibody staining for Ci155 (full-length Cubitus interruptus) and Engrailed, which confer anterior and posterior compartmental identities, respectively (Figure 2A). Immunofluorescence staining of wing discs with antibodies against DE-Cadherin and Discs large (Dlg) revealed no obvious cell polarity defect in the epithelium of the *mahj* mutant (Figure S2D). Compared with wild-type or homozygous mutants, apoptosis was not altered in homozygous mutant wing discs (unpublished data).

To explore the function of Mahjong, we used the mitotic recombination technique with the flipase (FLP)–flipase recombination target (FRT) system to generate mosaic tissues (Figure S2E) [24]. Using this approach, the same numbers of *mahj* homozygous mutant (*mahj* ^−/−^) and sibling wild-type cells were initially produced. In the mosaic wing discs, 72 h after clone induction, both *mahj* ^−/−^ and sibling wild-type clones were readily detected (Figure 2B). However, at 96 h, the size of the *mahj* ^−/−^ clones was clearly smaller than that of sibling wild-type clones (Figure 2C), and at 120 h, most of the *mahj* ^−/−^ clones had disappeared from the wing disc epithelium (Figure 2D). Immunofluorescence with anti-active Caspase 3 antibody revealed that *mahj* ^−/−^ clones adjacent to wild-type or *mahj^+/^* ^−^ cells frequently underwent apoptosis (Figure 2E–G), whereas *mahj* ^−/−^ clones that were not adjacent to wild-type or *mahj^+/^* ^−^ cells rarely did (Figure 2G). These observations suggest that the abutting or near-by wild-type or *mahj^+/^* ^−^ cells trigger the *mahj* ^−/−^ cells to undergo apoptosis. Although the majority of apoptotic cells were basally extruded from the disc epithelium, we found some apoptotic *mahj* ^−/−^ cells remaining within the epithelial layer (Figures S2F and S7B, arrows), suggesting that apoptosis was not caused by extrusion.

This type of competition between wild-type and mutant cells was first reported in *Minute* mutant flies [25]. *Minute* is a generic name given to genes encoding ribosomal proteins or other protein-synthesis components. Heterozygous *Minute* flies are viable but develop slowly, though their final body size is similar to that of wild-type flies. When surrounded by wild-type cells, however, *Minute* heterozygous mutant cells (*M/+*) undergo apoptosis and are eliminated from the epithelial monolayer. A similar competition for cell survival, termed cell competition, has also been reported between wild-type and other types of mutant cells [6],[7],[8]. Interestingly, we found that *mahj* ^−/−^ cells did not undergo apoptosis when they were surrounded by *M/+* cells (Figure S2G). Collectively, these findings indicate that *mahj* ^−/−^ clones are eliminated from the wild-type disc epithelium through the process of cell competition.

To examine the functional role of Mahjong/VprBP (hereafter referred to as Mahjong) in mammalian cells, we established MDCK cell lines that stably express Mahjong shRNA in a tetracycline-inducible manner (MDCK pTR Mahjong shRNA). At 48 h after tetracycline addition, 80%–90% of Mahjong was knocked down (Figure S3A). When Mahjong expression was knocked down, cells became slightly flattened but maintained their epithelial morphology and tight cell-cell adhesions (Figure S3B). Knockdown of Mahjong did not affect the expression level of mLgl2 or PKCζ (Figure S3C). When MDCK pTR Mahjong shRNA cells were cultured with tetracycline in a 3D culture system [26], they formed cysts with a regular lumen and well-defined F-actin-rich apical membrane domain (Figure S3D), and other cell polarity marker proteins, including PKCζ, β-catenin, and ZO-1, were properly localized (Figure S3D and S3E). These findings are consistent with the fly data (Figure S2D) and confirm that Mahjong does not play a crucial role in the establishment or maintenance of epithelial cell polarity.

**Figure 3:**
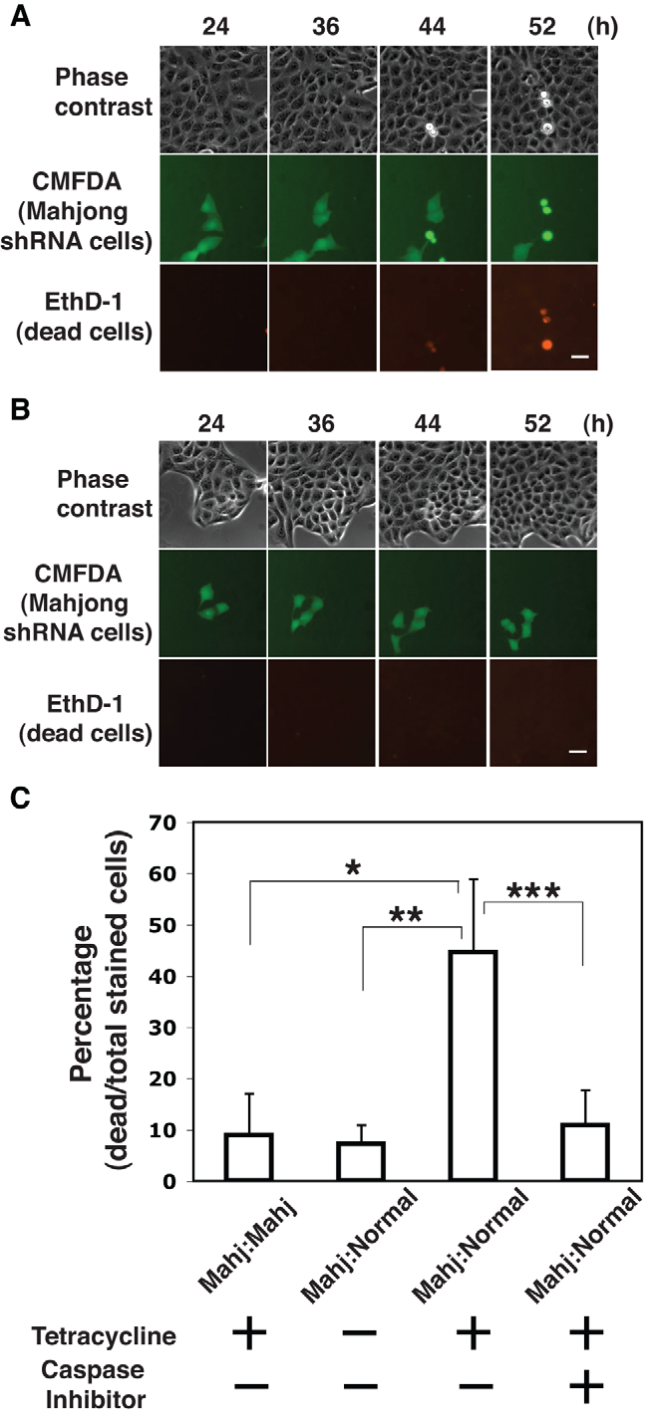
Knockdown of Mahjong expression induces cell competition in cultured MDCK epithelial cells. (A and B) MDCK pTR Mahjong shRNA cells were fluorescently labeled with green fluorescent dye CMFDA (green) and mixed with normal MDCK cells at a ratio of 1∶10, and cultured in the presence (A) or absence (B) of tetracycline with EthD-1 (red) for the indicated times. Images were extracted from a representative time-lapse analysis. Scale bar: 30 µm. (C) Quantification of cell death of MDCK pTR Mahjong shRNA cells. Fluorescently labeled MDCK pTR Mahjong shRNA cells were mixed with normal MDCK or MDCK pTR Mahjong shRNA cells, and cultured in the absence or presence of tetracycline or caspase inhibitor (Z-VAD-FMK) for 60 h. Frequency of cell death that occurred in labeled MDCK pTR Mahjong shRNA cells was analyzed for 30–60 cells in each experimental condition, and the results represent the means±SD of more than three independent experiments. *p<0.005; **p<0.001; ***p<0.02. Noted that all and only dead cells were apically extruded.

Next, we tested for involvement of Mahjong in cell competition in this cell culture system. MDCK pTR Mahjong shRNA cells were labeled with green fluorescent dye (CMFDA) and mixed with normal MDCK cells at a ratio of 1∶10. The mixture of cells was then cultured in the absence of tetracycline until the cells formed a monolayer. We then induced the expression of Mahjong shRNA with tetracycline and observed the fate of Mahjong-knockdown cells surrounded by normal cells using time-lapse microscopy (Vidoes S1, S2). To monitor cell death, we added ethidium homodimer-1 (EthD-1), which passes through the damaged plasma membrane of dead cells, binds to nucleic acids, and produces a red fluorescence. After 24–52 h of tetracycline addition, ∼45% of Mahjong shRNA cells died and were extruded from the apical surface of the monolayer (Figure 3A and 3C). Addition of a caspase inhibitor (Z-VAD-FMK) significantly suppressed both cell death and extrusion (Figure 3C), indicating that apical extrusion of Mahjong-knockdown cells resulted from apoptosis. This result is consistent with that from a previous report that apoptotic cells are apically extruded from a monolayer of MDCK cells [27]. Overexpression of exogenous Mahjong protein in Mahjong shRNA cells prevented their apical extrusion (Figure S4A and S4B), suggesting that apoptosis of Mahjong shRNA cells is indeed attributed to Mahjong-knockdown. In the absence of tetracycline, Mahjong shRNA cells did not die and were not extruded (Figure 3B and 3C and Video S3). Moreover, fluorescently labeled Mahjong-knockdown cells were not extruded when they were mixed with unlabeled Mahjong-knockdown cells (Figure 3C and Figure S5), suggesting that the apoptosis of Mahjong-knockdown cells depends on the presence of surrounding normal cells. Similarly, fluorescently labeled normal MDCK cells did not undergo apoptosis when surrounded by normal MDCK cells (unpublished data) [28]. Taken together, these results indicate that the death of Mahjong-knockdown cells occurs only when they are surrounded by normal cells. As far as we are aware, this is the first evidence showing that cell competition can occur in a mammalian cell culture system.

**Figure 4:**
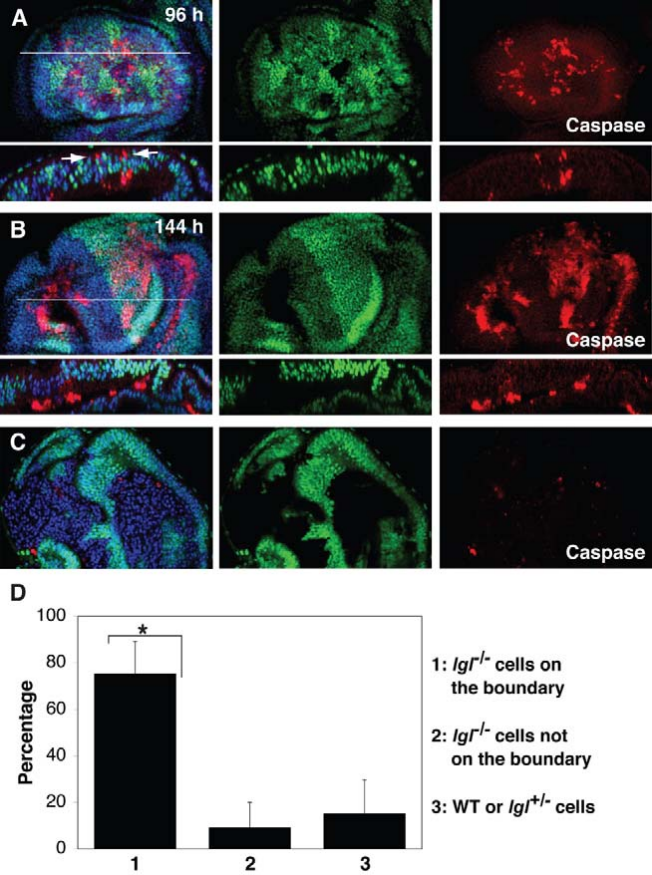
Loss of Lgl function induces cell competition in the *Drosophila* wing disc epithelium. (A and B) Wing discs with *lgl^4^* homozygous (lacking GFP), wild-type (expressing GFP strongly), and *lgl^4^* heterozygous clones (expressing GFP moderately) at 96 h (A) or 144 h (B) after clone induction. Note that most of the apoptotic *lgl^4^* homozygous clones (lacking GFP) were basally extruded. Upper panels: A Z-stack projection of 40 confocal images of a wing disc. Lower panels: Transverse sections of the white line. (A) Arrows indicate apoptotic *lgl* ^−/−^ cells remaining within the epithelial monolayer. (C) A wing disc where *lgl* ^−/−^ clones are surrounded by *Minute/+* heterozygous cells (expressing GFP) at 144 h after clone induction. (A–C) Apoptotic cells were labeled with anti-cleaved Caspase-3 antibody (red). Nuclei were stained with DAPI (blue). (D) Quantification of apoptotic cells in the *lgl* ^−/−^ mosaic wing discs at 96 h after clone induction, showing non-cell-autonomous apoptosis in *lgl* ^−/−^ cells. The results represent means±SD (*n* = 31 discs, *p<0.001).

Mammals harbor two Lgl homologues, mLgl1 and mLgl2. We observed that Mahjong was coimmunoprecipitated with mLgl1 as well as with mLgl2 (Figure S6). Because of potential functional redundancy between mLgl1 and mLgl2 [12], investigating the functional linkage between Lgl and Mahjong in mammalian cells is technically very difficult. We therefore examined wing imaginal discs of *Drosophila melanogaster*, where the function of Lgl has been intensively studied [9],[10],[13]. First, we used the FLP-FRT system to generate mosaic wing discs bearing *lgl^4^* (null mutant allele of *lgl*) homozygous mutant clones. By 96 h after clone induction, a number of *lgl* ^−/−^ cells abutting wild-type cells had become apoptotic and were basally extruded (Figure 4A), as previously reported for eye discs [13]. Some apoptotic cells stayed in the epithelial layer (Figure 4A and Figure S7C, arrows), however, suggesting that the apoptosis was not caused by basal extrusion. At 144 h, most of the *lgl* ^−/−^ clones had been basally extruded from the epithelium (Figure 4B), frequently associated with indentations of the epithelial layer (Figure 4B). In contrast, when surrounded by *M/+* cells, *lgl* ^−/−^ clones survived and showed an overgrowth phenotype (Figure 4C). Quantification analyses revealed that apoptosis was mostly detected in mutant cells that were at the clone boundary (Figure 4D), suggesting that the presence of the abutting or nearby wild-type or *lgl^+/^* ^−^ cells triggers apoptosis of *lgl* ^−/−^ cells.

**Figure 5:**
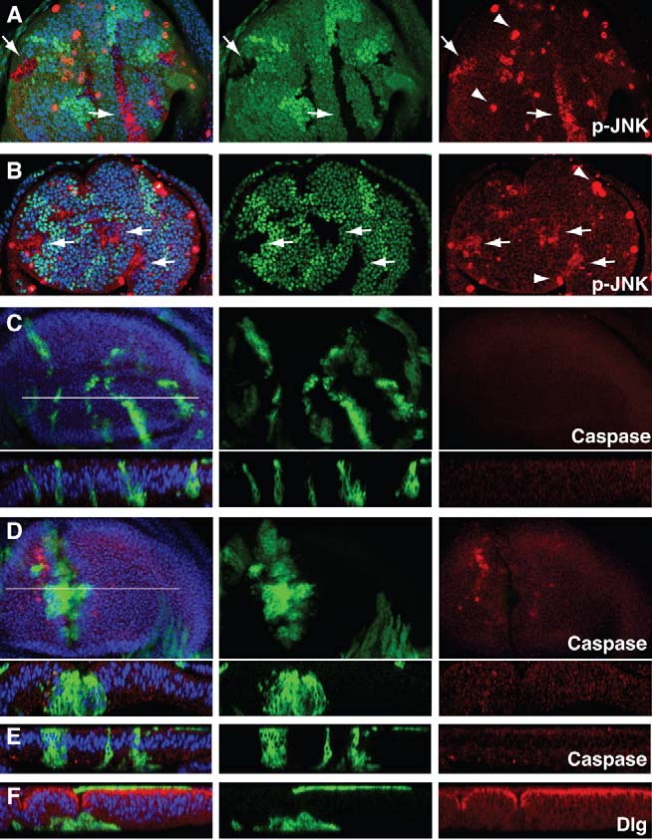
JNK is involved in the apoptosis of *mahj* ^−/−^ and *lgl* ^−/−^ clones. (A and B) Wing discs with *mahj* ^−/−^ (A) or *lgl* ^−/−^ (B) clones (lacking GFP), wild-type clones (expressing GFP strongly), and heterozygous clones (expressing GFP moderately) at 96 h after clone induction. Arrows indicate phospho JNK (p-JNK)-positive *mahj* ^−/−^ or *lgl* ^−/−^ cells. Some strong punctate p-JNK signals (arrowheads) are derived from cells within the peripodial layer where endogenous JNK activity is upregulated [39]. (C–F) The mosaic analysis with a repressible cell marker (MARCM) system was used to overexpress UAS constructs in either *mahj* ^−/−^ clones (C and E) or *lgl* ^−/−^ clones (D and F), and homozygous mutant clones are marked by the expression of GFP. (C and D) Overexpression of *puc* in *mahj* ^−/−^ (C) or *lgl* ^−/−^ (D) clones at 120 h after clone induction. Upper panels: A Z-stack projection of 40 confocal images of a wing disc. Lower panels: Transverse sections of the white line. (E and F) Transverse section of a wing disc with *mahj* ^−/−^ (E) or *lgl* ^−/−^ (F) MARCM clones overexpressing *p35* at 144 h after clone induction. Anti-phospho JNK (A and B), anti-cleaved Caspase-3 (C–E), and anti-Dlg (F) antibodies were used for immunostaining. (A–F) Nuclei were stained with DAPI (blue).

We then further analyzed the molecular mechanisms of apoptosis of *mahj* ^−/−^ and *lgl* ^−/−^ cells using the mosaic analysis with a repressible cell marker (MARCM) system (Figure S7A), which allowed us to express genes of interest in the mutant cells. We first confirmed that apoptosis of *mahj* ^−/−^ and *lgl* ^−/−^ cells occurred in this system (Figure S7B and S7C), as it did in the FLP-FRT system (Figures 2E, 2F, 4A, and 4B). Expression of exogenous Mahj or Lgl alleviated all phenotypes, including apoptosis and basal extrusion, in *mahj* ^−/−^ and *lgl* ^−/−^ clones, respectively (Figure S7D and S7E). Activation of the JNK pathway has been shown to be involved in cell competition observed with various mutations [7],[8],[29],[30]. Indeed, we observed that JNK was ectopically activated in both *mahj* ^−/−^ and *lgl* ^−/−^ clones (Figures 5A and 5B, arrows, and S7F). *puckered* (*puc*) is a gene encoding a protein phosphatase that dephosphorylates and inactivates activated JNK. Overexpression of *puc* strongly suppressed the apoptosis of both *mahj* ^−/−^ and *lgl* ^−/−^ clones (Figure 5C and 5D). Similarly, overexpression of p35, an anti-apoptotic protein, suppressed the apoptosis in both *mahj* ^−/−^ and *lgl* ^−/−^ clones (Figure 5E, and unpublished data). These results indicate that JNK is involved in the apoptosis of *mahj* ^−/−^ and *lgl* ^−/−^ clones. We also found that addition of JNK inhibitor SP600125 significantly suppressed cell death and apical extrusion of MDCK Mahjong shRNA cells that were surrounded by normal MDCK cells (Figure S8), suggesting that the involvement of JNK in Mahjong-mediated cell competition is evolutionarily conserved.

In addition to these cell competition phenotypes, we also observed apoptosis-independent basal extrusion of *mahj* ^−/−^ and *lgl* ^−/−^ cells. In the wing disc epithelium, when mutations of *mahj* or *lgl* were induced in a mosaic manner, some basally extruded *mahj* ^−/−^ or *lgl* ^−/−^ cells were not stained with anti-active Caspase 3 antibody (Figure S7B and S7C, arrowheads), though the majority of basally extruded cells were stained. Comparable basal extrusion of *mahj* ^−/−^ or *lgl* ^−/−^ cells was not seen in the epithelium of *mahj* or *lgl* homozygous animals (Figure S2D, and unpublished data), indicating that the apoptosis-independent basal extrusion occurs only when mutant clones are surrounded by wild-type cells. In addition, although overexpression of *puc* or p35 suppressed the apoptosis of *mahj* ^−/−^ or *lgl* ^−/−^ clones (Figure 5C–E), it did not fully block basal extrusion (Figure 5C–F). These findings indicate that *mahj* ^−/−^ *and lgl* ^−/−^ clones can be basally extruded in either an apoptosis-dependent or -independent manner. However, at present it is not clearly understood how the fate of the clones, whether they die or not prior to basal extrusion, is determined.

**Figure 6:**
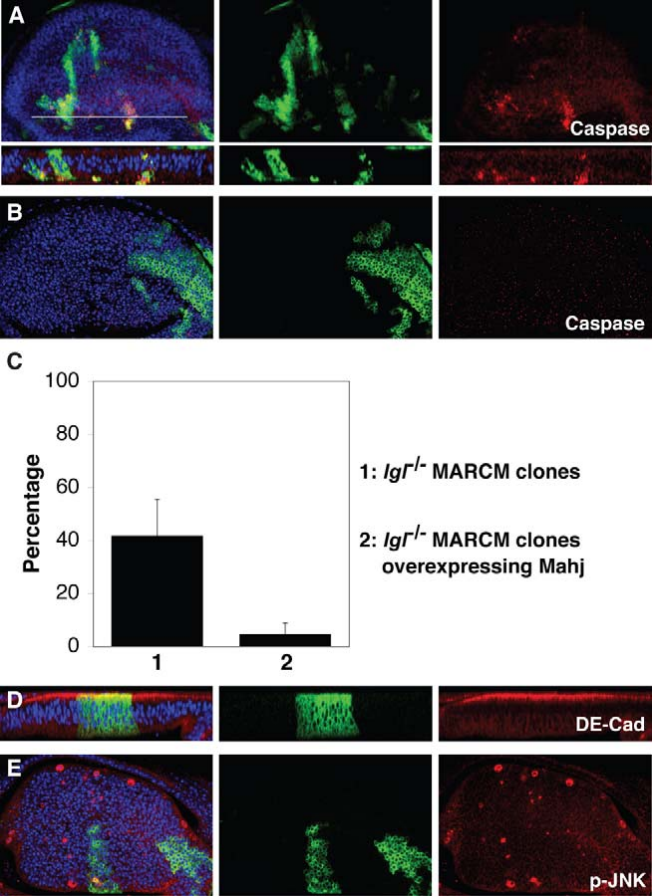
Mahjong acts as a downstream target of Lgl in cell competition and basal cell extrusion. The mosaic analysis with a repressible cell marker (MARCM) system was used to overexpress UAS constructs in either *mahj* ^−/−^ clones (A) or *lgl* ^−/−^ clones (B, D, and E). Homozygous mutant clones are marked by the expression of GFP. (A) *mahj* ^−/−^ MARCM clones overexpressing Lgl at 96 h after clone induction. (B, D, and E) *lgl* ^−/−^ MARCM clones overexpressing Mahj at 144 h (B and D) or 96 h (E) after clone induction. (C) Quantification of apoptotic cells in the *lgl* ^−/−^ mosaic wing discs with or without overexpression of Mahj. The percentage of apoptosis occurring in *lgl* ^−/−^ cells on the boundary with wild-type cells was examined. The results represent means±SD (*n* = 13 discs, *p<0.001). (D) A transverse section. Anti-cleaved Caspase-3 (A and B), anti-DE-Cadherin (D), and anti-phospho JNK (E) antibodies were used for immunostaining. (A, B, D, and E) Nuclei were stained with DAPI (blue).

Finally, we investigated the epistatic relationship between *lgl* and *mahjong*. We generated *mahj* ^−/−^ and *lgl* ^−/−^ MARCM clones overexpressing Lgl and Mahj, respectively. Overexpression of Lgl did not affect the phenotype of *mahj* ^−/−^ clones; the cells underwent apoptosis and were basally extruded (Figure 6A). In contrast, overexpression of Mahj strongly suppressed both the apoptosis and basal extrusion of *lgl* ^−/−^ clones (Figure 6B–D); the cells remained within the epithelial layer with normal apicobasal polarity. Mahj overexpression allowed survival of *lgl* ^−/−^ clones to adulthood (Figure S9). We also found that overexpression of Mahj suppressed the JNK activation in *lgl* ^−/−^ clones (Figure 6E). Overexpression of Mahj in wild-type clones did not induce apoptosis nor affect cell growth (Figure S10A–C). Scribble is another tumor suppressor protein involved in cell competition; *scribble* ^−/−^ cells undergo apoptosis when surrounded by wild-type cells [8]. Overexpression of Mahj in *scribble* ^−/−^ clones did not suppress apoptosis of *scribble* ^−/−^ cells in a monolayer of wild-type wing disc cells (Figure S11A). Furthermore, apoptosis of *lgl* ^−/−^ cells was not suppressed by overexpression of dMyc (Figure S11B). These data suggest that the effect of Mahj overexpression on apoptosis is specific to *lgl* ^−/−^ clones. Collectively, these results indicate that Mahjong acts downstream of Lgl and is involved in Lgl-mediated cell competition.

In this study, we have identified Mahjong as an evolutionarily conserved Lgl-binding protein. We have demonstrated that *lgl* ^−/−^ or *mahj* ^−/−^ cells undergo apoptosis when surrounded by wild-type cells and that apoptosis of *lgl* ^−/−^ cells is alleviated by overexpression of Mahj. Loss of Mahjong function does not induce detectable cell polarity defects, suggesting that Mahjong does not function with Lgl in the establishment of cell polarity. It is plausible that Lgl positively regulates the function of Mahjong affecting molecules or signaling pathways that set the competitiveness of cells. However, at present, it is not clear how Lgl interacts with Mahjong in cell competition and what molecules/signaling pathways are regulated by Lgl/Mahjong. Answers to these questions would increase our understanding of the interactions between normal and transformed cells and might lead to novel cancer therapies.

## Materials and Methods

### Antibodies, Plasmids, and Materials

Rabbit polyclonal anti-VprBP/Mahjong antibody was obtained from Shanghai Genomics (Shanghai, China). Mouse anti-Myc (4A6) antibody was purchased from Upstate (Charlottesville, VA). Mouse anti-FLAG (M2) and peroxidase-conjugated mouse anti-FLAG (M2) antibodies were from Sigma-Aldrich (St. Louis, MO). The former was used for immunoprecipitation and the latter for Western blotting. Mouse anti-Llgl2 and rabbit anti-ZO-1 antibodies were purchased from Abnova (Taipei, Taiwan) and Zymed (South San Francisco, CA), respectively. Rabbit anti-PKCζ antibody was from Santa Cruz Biotechnology (Santa Cruz, CA). Mouse anti-β-catenin and rabbit anti-phospho-histone H3 (PH3) antibodies were purchased from BD Biosciences (Lexington, KY). Mouse anti-GFP antibody was obtained from Roche Diagnostics (Mannheim, Germany) and was used for immunoprecipitation and Western blotting except in Figure S1D, where rabbit anti-GFP antibody (Invitrogen; Carlsbad, CA) was used for Western blotting. Mouse anti-GAPDH antibody was from Chemicon International (Temecula, CA). Mouse anti-Engrailed (4D9), mouse anti-β-galactosidase (40-1a), rat anti-DE-Cadherin (DCAD2), and mouse anti-Dlg (4F3) antibodies were obtained from Developmental Studies Hybridoma Bank. Rat monoclonal antibody recognizing only Ci-155 (2A1) was a gift from R. Holmgren and T.B. Kornberg. Rabbit anti-cleaved Caspase-3 antibody (Asp 175) was from Cell Signaling Technology (Danvers, MA). Anti-phospho JNK antibody (pTPpY) was from Promega (Madison, WI). All antibodies were used at dilution of 1∶1000 for Western blotting and 1∶100 for immunofluorescence except as follows for immunofluorescence: anti-Ci-155 (1∶2), anti-Engrailed (1∶2), anti-β-galactosidase (1∶50), anti-DE-cadherin (1∶30), anti-Dlg (1∶50), and anti-PH3 (1∶200). DAPI (4′, 6-diamidino-2-phenylindole dihydrochloride) was from Invitrogen. BrdU labeling was carried out as previously described [31]. SYPRO Ruby protein gel staining solution was obtained from Invitrogen.

pEGFP-VprBP/Mahjong was constructed by digestion of a human VprBPα EST clone (IMAGE clone 4853730, Geneservice Ltd.) with SacI/EcoRI and ligation into SacI/EcoRI sites of pEGFP-C3. To construct pEGFP-VprBP/Mahjong-N (hereafter referred to as Mahjong), pEGFP-Mahjong-M, and pEGFP-Mahjong-C, the cDNAs of the respective Mahjong portion were amplified by PCR with the following primers: Mahjong-N, 5′-GGCCTCGAGCCATGACTACAGTAGTGGTACATGTGG-3′ and 5′-GCAGAATTCGATCATTGGTCTGCATCTGTGATGGGC-3′; Mahjong-M, 5′-GGCCTCGAGCGCCCATCACAGATGCAGACCAAATCC-3′ and 5′-GCAGAATTCGATCAACAGTTATAGCTGGCCTCCTCC-3′; Mahjong-C, 5′-GGCCTCGAGCGGAGGAGGCCAGCTATAACTGT-3′ and 5′-GCAGAATTCGAGATGGCTCCTCACTCATTCAGAG-3′. These cDNAs were cloned into XhoI/EcoRI sites of pEGFP-C1. To construct pcDNA4/TO/GFP-Mahjong, the cDNA of Mahjong was excised from pEGFP-Mahjong (XhoI/EcoRI) and, after blunting the ends, inserted into pcDNA4/TO/GFP (XhoI/EcoRI blunt-ended). To construct pSUPERIOR.neo+gfp Mahjong shRNA, Mahjong shRNA oligonucleotides (5′-GATCCCCAGAGCTGCTTCTGTTGATATTCAAGAGATATCAACAGAAGCAGCTCTTTTTTC-3′ and 5′-TCGAGAAAAAAGAGCTGCTTCTGTTGATATCTCTTGAATATCAACAGAAGCAGC-3′) were cloned into BglII/XhoI sites of pSUPERIOR.neo+gfp (Oligoengine, Seattle, WA). pFLAG-CMV2-mLgl1, pEGFP-mLgl2-WT, pcDNA-FLAG-mLgl2-WT, pcDNA-FLAG-mLgl2-N, and pcDNA-FLAG-mLgl2-C was described previously [26].

### Cell Culture, Immunofluorescence, Immunoprecipitation, and Western Blotting

MDCK and MCF-7 cells were cultured as previously described [32]. To obtain MCF-7 cells stably expressing GFP-mLgl2, MCF-7 cells were transfected with pEGFP-mLgl2 using Lipofectamine 2000 (Invitrogen), and stably transfected cells were selected in medium containing 800 µg ml^−1^ of G418 (Calbiochem, Darmstadt, Germany). To establish MDCK cell lines that stably express Mahjong shRNA in a tetracycline-inducible manner (MDCK pTR Mahjong shRNA), MDCK pTR cells [26] were transfected with pSUPERIOR.neo+gfp Mahjong shRNA using Metafectene Pro (Biontex, München, Germany), followed by selection in medium containing 5 µg ml^−1^ of blasticidin (Invitrogen) and 800 µg ml^−1^ of G418. To establish MDCK cell lines that stably express Mahjong shRNA and GFP-Mahjong in a tetracycline-inducible manner (MDCK pTR Mahjong shRNA+GFP-Mahjong), MDCK pTR Mahjong shRNA cells were transfected with pcDNA4/TO/GFP-Mahjong, followed by selection in medium containing 5 µg ml^−1^ of blasticidin, 400 µg ml^−1^ of zeocin (Invitrogen), and 800 µg ml^−1^ of G418. Knockdown of Mahjong was analyzed by Western blotting after 48 h of incubation with tetracycline (2 µg ml^−1^). HEK 293 cells were cultured and transfected as previously described [33]. *Drosophila* S2R+ cells were cultured and transfected with Fugene HD (Roche) as previously described [34]. Immunoprecipitation and Western blotting were performed as previously described [35].

Immunofluorescence of cells cultured on serum-coated glass coverslips was performed as previously described [35]. MDCK pTR Mahjong shRNA cells were cultured in collagen gels as previously described [26] and were processed for immunofluorescence staining as previously described [36]. Immunofluorescent images were analyzed by confocal microscopy, if not otherwise indicated. To obtain confocal images of cultured cells, we used a Leica TCS SPE confocal microscope and Leica Application Suite (LAS) software. To obtain phase-contrast images, we used a Leica DMIRB microscope with a Hamamatsu C4742-95 Orca camera. Images were captured and analyzed with Openlab (Improvision) and ImageJ 1.36b (National Institutes of Health). For analyses of imaginal discs of *Drosophila*, larvae were chosen at the given time after clone induction, and dissected tissues were fixed in 4% formaldehyde at room temperature for 15–30 min. All subsequent steps for immunostaining were performed according to standard procedures for confocal microscopy [37]. All samples were counterstained with DAPI for visualization of DNA. Images were acquired with a Zeiss LSM 510 confocal microscope.

### LC-MS/MS Procedure

mLgl2-interacting protein gel bands were excised and in-gel digested with trypsin. The digest mixtures were separated by nanoscale liquid chromatography (LC Packings) on reverse phase C18 column (150×0.075 mm ID, flow rate 0.15 ml/min). The eluate was introduced directly into a Q-STAR Pulsar-i-hybrid quadruple-time-of-flight mass spectrometer (MDS Sciex). The spectra were searched against a NCBI non-redundant database with MASCOT MS/MS Ions search (Matrixscience).

### Time-Lapse Microscopy

MDCK pTR Mahjong shRNA cells or MDCK pTR Mahjong shRNA+GFP-Mahjong cells were fluorescently labeled with CellTracker dye CMFDA (green) or CMTPX (red) (Invitrogen) according to the manufacturer's instructions. Labeled cells were then trypsinized and mixed with MDCK or MDCK pTR Mahjong shRNA cells at a ratio of 1∶10. Cells were plated at a density of 4×10^5^ cells in 35 mm glass-bottom culture dishes (MatTek Corporation, Ashland, MA) for all experiments. Mixed cells were incubated for 24 h in medium with or without tetracycline, and then analyzed by time-lapse microscopy where EthD-1 (red) or SYTOX Blue dye (blue) (Invitrogen) was added to the medium according to the manufacturer's instructions for monitoring of cell death. Where indicated, cells were analyzed in the presence of 100 µM caspase inhibitor Z-VAD-FMK (Calbiochem) or 5 µM JNK inhibitor SP600125 (Invitrogen). To obtain time-lapse images, we used a Zeiss Axiovert 200M microscope with a Ludl Electronic Products Biopoint Controller and a Hamamatsu C4742-95 Orca camera (Hamamatsu). Images were captured and analyzed with Openlab or Volocity software (Improvision).

### Generation and Characterization of the *mahjong^1^* Allele

The P-element insertion fly, *w^1118^*; *P{GT1}GT-000304*, is viable and exhibits a normal phenotype. Excisions of GT-000304 were tested for complementation of the deletion *Df(2R)XE-2900*. The flanking genomic region of the GT-000304 insertion site of those failing to complement *Df(2R)XE-2900* was sequenced. *mahj^1^* was a deletion of 1,633 bp including exons 10 and 11, part of exon 9, and the 3′-UTR of CG10080. To alleviate the lethality of *mahj* homozygotes we used the following genotype: *mahj^1^/mahj^1^*; *act-Gal4/UAS-mahj*.

### Fly Strains and Crosses

*Drosophila* stocks were maintained by standard methods at 25°C. *mahj^1^* was recombined to the FRT42D chromosome. To generate a *UAS-mahj* transgenic fly, a full-length cDNA clone of *mahj* (obtained from Drosophila Genomics Resource Center, IN) was amplified by PCR, sequenced, and subcloned into pUASP for *Drosophila* transgene [38]. Transgenic lines of *pUASP:mahj* were generated by standard methods. *mahj* and *lgl* mosaic mutant clones were generated by the FLP-FRT-mediated mitotic recombination system [24] in the following genotypes: *hsFLP*; *FRT42D mahj^1^/FRT42D ubi-EGFP* or *hsFLP*; *lgl^4^ FRT40A/hGFP FRT40A*. To block apoptosis in *mahj* and *lgl* MARCM clones, we used the following genotypes: *UAS-CD8-GFP*, *hsFLP*; *FRT42D mahj^1^/FRT42D ubi-Gal80*; *tubP-Gal4/UAS-p35* or *hsFLP*; *lgl^4^ FRT40A/tubP-Gal80 FRT40A*; *tubP-Gal4*, *UAS-GFP/UAS-p35*. To block JNK activation in *mahj* and *lgl* MARCM clones, we used the following genotypes: *UAS-CD8-GFP*, *hsFLP*; *FRT42D mahj^1^/FRT42D ubi-Gal80*; *tubP-Gal4/UAS-puc* or *hsFLP*; *lgl^4^ FRT40A/tubP-Gal80 FRT40A*; *tubP-Gal4*, *UAS-GFP/UAS-puc*. To express Lgl in *mahj* MARCM clones, we used the following genotypes: *UAS-CD8-GFP*, *hsFLP*; *FRT42D mahj^1^/FRT42D ubi-Gal80*; *tubP-Gal4/UAS-lgl*. To express Mahjong in *lgl* MARCM clones, we used the following genotypes: *hsFLP*; *lgl^4^ FRT40A/tubP-Gal80 FRT40A*; *tubP-Gal4*, *UAS-GFP/UAS-mahjong*. To express Mahjong in *mahj* MARCM clones, we used the following genotypes: *UAS-CD8-GFP*, *hsFLP*; *FRT42D mahj^1^/FRT42D ubi-Gal80*; *tubP-Gal4/UAS-mahj*. To express Lgl in *lgl* MARCM clones, we used the following genotypes: *hsFLP*; *lgl^4^ FRT40A/tubP-Gal80 FRT40A*; *tubP-Gal4*, *UAS-GFP/UAS-lgl*. For the *Minute* technique in wing discs, mosaic clones were generated in the following genotype: *hsFLP*; *FRT42D mahj^1^/FRT42D arm-lacZ M(2)92* and *hsFLP*; *lgl^4^ FRT40A/M(2)25A ubi-GFP FRT40A*. To express dMyc in *lgl* MARCM clones, we used the following genotypes: *hsFLP*; *lgl^4^ FRT40A/tubP-Gal80 FRT40A*; *tubP-Gal4*, *UAS-GFP/UAS-dMyc*. To express Mahjong in *scrib* MARCM clones, we used the following genotypes: *hsFLP*; *act-Gal4*, *UAS-GFP/UAS-mahjong*; *FRT82B scrib^1^/FRT82B tubP-Gal80*. *UAS-mahj* overexpression clones were generated by the Flip-out Gal4 system in the following genotypes: *hsFLP*; *UAS-mahjong/act>CD2>Gal4*, *UAS-GFP*. *UAS-GFP* overexpression clones were generated by the Flip-out Gal4 system in the following genotypes: *hsFLP*; *act>CD2>Gal4*, *UAS-GFP*.

### Generation of Mutant Clones

All fly crosses were carried out at 25°C according to standard procedures. Mutant clones were generated by mitotic recombination with the FLP/FRT system by X-chromosome heat-shock-inducible flipase (hsFLP) [24]. Clones were marked with ubi-EGFP, histone-GFP (hGFP), or arm-lacZ. To obtain mosaic mutant clones in wing discs, we heat shocked first- or second-instar larvae for 1 h at 37°C. For generation of Flip-out Gal4 overexpression clones of UAS-mahjong or UAS-GFP in wing discs, second-instar larvae were heat-shocked for 15 min at 37°C. The size of each clone expressing UAS-mahjong or UAS-GFP in wing discs was measured with the pixel measurement function of ImageJ.

### Statistical Analysis

Student's *t* tests assuming paired variances were performed for statistical analysis.

## Supporting Information

Figure S1 (A) A schematic illustrating the mLgl2 and VprBP constructs used (red, WD repeats; green, phosphorylation sites; purple, Lis1 homology motif domain). (B) Interaction of VprBP with the C-terminus of mLgl2. The full-length (FLAG-mLgl2-WT), N-terminus (FLAG-mLgl2-N), or C-terminus (FLAG-mLgl2-C) of mLgl2 was coexpressed with GFP-VprBP in human embryonic kidney (HEK) 293 cells. Immunoprecipitation was performed with anti-FLAG antibody, followed by Western blotting with anti-FLAG and anti-GFP antibodies. (C) Interaction of mLgl2 with the C-terminus of VprBP. The full-length (GFP-VprBP-WT), N-terminus (GFP-VprBP-N), middle part (GFP-VprBP-M), or C-terminus (GFP-VprBP-C) of VprBP was coexpressed with FLAG-mLgl2-WT in HEK293 cells. Immunoprecipitation was performed with anti-FLAG antibody, followed by Western blotting with anti-FLAG and anti-GFP antibodies. The arrowhead indicates the position of IgG heavy chains. (B and C) In human, two isoforms of VprBP are produced from the *VprBP* gene by alternative splicing. The shorter form consists of 1,058 amino acids and the longer form of 1,507 amino acids. The difference in length is due to an insertion of 449 amino acids after residue 225 in the long form. In these experiments, we used GFP-tagged shorter VprBP isoform. (D) Interaction of Lgl with *Drosophila* Mahjong protein. Myc-tagged Lgl was coexpressed with GFP or GFP-Mahjong in S2R+ cells, and immunoprecipitation was performed with anti-GFP antibody, followed by Western blotting with anti-Myc and anti-GFP antibodies. Mouse and rabbit anti-GFP antibodies were used for immunoprecipitation and Western blotting, respectively. The arrow and arrowhead indicate the positions of GFP-Mahjong and GFP, respectively.
(0.45 MB TIF)

Figure S2 (A) Schematic representation of the genome region containing the *mahjong* (CG10080) locus. Predicted genes are indicated as boxes. The P-element insertion site of GT-000304 and the extent of the *mahj^1^* deletion are indicated. (B) A line graph showing the growth defect of the homozygous *mahj^1^* mutant larvae and the trans-heterozygous mutant larvae with *Df(2R)XE-2900*. Each value represents the percentage of pupariated animals on the indicated days after egg laying relative to the final number of pupae. *Df(2R)XE-2900*: a mutant of chromosomal deficiency in which the entire coding region of *mahj* is deleted. (C) Comparison of the heterozygous *mahj^1^* control female (left) with the homozygous *mahj^1^* mutant female rescued by expression of UAS-Mahjong under the control of actin-GAL4 (right). (D) Transverse sections of a wing disc of homozygous *mahj^1^* larva that were immunostained with anti-DE-Cadherin and anti-Dlg antibodies. (E) Wing discs with wild-type (lacking GFP) and GFP-expressing wild-type clones at 72 h, 96 h, and 120 h (left to right) after clone induction. (F) Transverse sections of a wing disc with *mahj* ^−/−^ (lacking GFP) and wild-type clones (expressing GFP strongly) at 96 h after clone induction. The arrow indicates an apoptotic *mahj* ^−/−^ cell remaining within the epithelial monolayer. (G) A wing disc where *mahj* ^−/−^ clones are surrounded by *Minute/+* heterozygous cells (expressing β-gal, green) at 144 h after clone induction. (D, F, and G) Nuclei were stained with DAPI (blue). (F and G) Apoptotic cells were labeled with anti-cleaved Caspase-3 antibody (red).
(1.06 MB TIF)

Figure S3 **Characterization of the effect of Mahjong knockdown in MDCK epithelial cells.** (A) Establishment of MDCK cell lines that stably express Mahjong shRNA in a tetracycline-inducible manner. Parental MDCK or MDCK pTR Mahjong shRNA cells (clone 3 or 4) were cultured with or without tetracycline for 48 h, and cell lysates were analyzed by Western blotting with anti-Mahjong or anti-GAPDH antibody. Note that comparable phenotypes were observed in cell polarity and cell competition with clones 3 and 4. (B) Effect of Mahjong knockdown on morphology in MDCK cells. MDCK pTR Mahjong shRNA cells were cultured with or without tetracycline for 48 h and were analyzed by phase-contrast microscopy. (C) Knockdown of Mahjong does not affect the expression of mLgl2 or PKCζ. Parental MDCK or MDCK pTR Mahjong shRNA cells were cultured with or without tetracycline for 48 h, and cell lysates were analyzed by Western blotting with anti-mLgl2 or anti-PKCζ antibody. (D and E) Immunofluorescence analyses of cell polarity markers in MDCK Mahjong shRNA cell cysts. MDCK pTR Mahjong shRNA cells were seeded in collagen gels and incubated with or without tetracycline for 11 d. Immunostaining was performed with anti-PKCζ antibody and TRITC-labeled phalloidin (D) or with anti-β-catenin and anti-ZO-1 antibodies (E). (B, D, and E) Scale bars: 10 µm.
(0.30 MB TIF)

Figure S4 **Overexpression of Mahjong alleviates the cell competition phenotype in MDCK Mahjong shRNA cells.** (A) Establishment of MDCK pTR Mahjong shRNA+GFP-Mahjong cells that stably express Mahjong shRNA and GFP-tagged human Mahjong in a tetracycline-inducible manner. Because of mismatch of a base pair, expression of Mahjong shRNA does not knock down exogenously expressed human Mahjong. MDCK pTR Mahjong shRNA+GFP-Mahjong cells were cultured with or without tetracycline for 48 h, and cell lysates were analyzed by Western blotting with anti-Mahjong or anti-GFP antibody. Arrowhead, arrows, and asterisks indicate the positions of endogenous Mahjong protein, exogenously expressed GFP-Mahjong protein, and its degradation products, respectively. Note that MDCK cells predominantly express the longer Mahjong isoform and that the GFP-tagged shorter isoform of human Mahjong is exogenously expressed. This result therefore suggests that the shorter Mahjong isoform can substitute for the longer isoform in cell competition. (B) Effect of overexpression of exogenous Mahjong on cell death and apical extrusion of MDCK pTR Mahjong shRNA cells that are surrounded by normal MDCK cells. Fluorescently labeled MDCK pTR Mahjong shRNA cells or MDCK pTR Mahjong shRNA+GFP-Mahjong cells were mixed with normal MDCK cells, and cultured in the presence of tetracycline for 60 h. Frequency of cell death that occurred in fluorescently labeled cells was analyzed for 50–120 cells in each experimental condition. Values are expressed as a ratio relative to MDCK pTR Mahjong shRNA cells, and the results represent the means±SD of three independent experiments. *p<0.005. Note that all and only dead cells were apically extruded.
(0.17 MB TIF)

Figure S5 **MDCK pTR Mahjong shRNA cells were fluorescently labeled with CMTPX (red), mixed with MDCK pTR Mahjong shRNA cells at a ratio of 1∶10, and cultured in the presence of tetracycline for the indicated times.** Images were extracted from a representative time-lapse analysis. CMTPX was used in this experiment because MDCK pTR Mahjong shRNA cells express a low level of GFP. Scale bars: 30 µm.
(0.29 MB TIF)

Figure S6 **Mahjong interacts with both mLgl1 and mLgl2.** GFP-Mahjong was coexpressed with FLAG-mLgl1 or FLAG-mLgl2 in HEK293 cells, and immunoprecipitation was performed with anti-FLAG antibody, followed by Western blotting with anti-FLAG and anti-GFP antibodies.
(0.25 MB TIF)

Figure S7 **Analyses of** ***mahj*** **^−/−^ or** ***lgl*** **^−/−^ clones with the MARCM system expressing UAS-GFP.** (A–C) Wild-type (A), *mahj* ^−/−^ (B), or *lgl* ^−/−^ (C) MARCM clones at 96 h after clone induction. (B and C) Arrows indicate apoptotic *mahj* ^−/−^ (B) or *lgl* ^−/−^cells (C) remaining within the epithelial layer. Arrowheads indicate basally extruded *mahj* ^−/−^ (B) or *lgl* ^−/−^cells (C) that were not stained with anti-active Caspase 3 antibody. (D) *mahj* ^−/−^ MARCM clones overexpressing Mahj 120 h after clone induction. (E) *lgl* ^−/−^ MARCM clones overexpressing Lgl at 120 h after clone induction. (F) A wild-type wing disc immunostained with anti-p-JNK antibody. Apoptotic cells were labeled with anti-cleaved Caspase-3 antibody (red), except in (F) where anti-p-JNK antibody (red) was used for immunostaining. (B and C) A Z-stack projection of 40 confocal images of a wing disc. (A–F) Nuclei were stained with DAPI (blue).
(2.01 MB TIF)

Figure S8 **Effect of JNK inhibitor on Mahjong knockdown-mediated cell competition in MDCK cells.** Fluorescently labeled MDCK pTR Mahjong shRNA cells were mixed with normal MDCK cells, and cultured with tetracycline in the absence or presence of JNK inhibitor (SP600125) for 60 h. Frequency of cell death that occurred in labeled MDCK pTR Mahjong shRNA cells was analyzed for 30–60 cells in each experimental condition. Values are expressed as a ratio relative to those in the absence of JNK inhibitor, and the results represent the means±SD of three independent experiments. *p<0.05. Note that all and only dead cells were apically extruded.
(0.08 MB TIF)

Figure S9 **Mahj overexpression allows survival of** ***lgl*** **^−/−^ clones to adulthood.** *lgl* ^−/−^ MARCM clones expressing UAS-GFP without (left) or with (right) UAS-mahj in the pharate adult. Note that GFP-positive *lgl* ^−/−^ clones are observed within the wing (outlined with a white line) in the right panel, but not in the left panel.
(0.20 MB TIF)

Figure S10 **Overexpression of Mahj in wild-type cells does not induce apoptosis nor affect cell growth.** (A and B) Wing discs with Mahj overexpression clones (green) at 96 h after clone induction. The Mahj overexpression clones were induced by hsFLP-Flip-out Gal4. (A) Apoptotic cells were labeled with anti-cleaved Caspase-3 antibody (red). (B) BrdU incorporation was detected by anti-BrdU antibody (red). (A and B) Nuclei were stained with DAPI (blue). (C) Quantification of the size of clones overexpressing UAS-mahjong or UAS-GFP in wild-type wing discs at 48 h after heat-shock. The results represent means±SD (*n* = 30 discs for each experimental condition).
(0.91 MB TIF)

Figure S11 **Inhibitory effect of Mahjong overexpression on apoptosis is specific to** ***lgl*** **^−/−^ clones.** The mosaic analysis with a repressible cell marker (MARCM) system was used to overexpress UAS constructs in either *scrib* ^−/−^ clones (A) or *lgl* ^−/−^ clones (B), and homozygous mutant clones are marked by the expression of GFP. (A) Overexpression of Mahj in *scrib* ^−/−^ clones at 72 h after clone induction. (B) Overexpression of dMyc in *lgl* ^−/−^ clones at 120 h after clone induction. Upper panels: A Z-stack projection of 40 confocal images of a wing disc. Lower panels: Transverse sections of the white line. Anti-cleaved Caspase-3 antibody was used for immunostaining (red). Nuclei were stained with DAPI (blue).
(0.89 MB TIF)

Video S1 **This video shows that Mahjong shRNA cells undergo apoptosis when they are cultured with normal cells in the presence of tetracycline.** MDCK pTR Mahjong shRNA cells were fluorescently labeled with CMFDA (green), mixed with normal MDCK cells at a ratio of 1∶10, and cultured in the presence of tetracycline. Time-lapse images were captured at 10 min intervals for 60 h, with fluorescent images captured every 30 min. (http://www.ucl.ac.uk/lmcb/images/Movie1.mov)
(4.67 MB MOV)

Video S2 **This is another video showing that Mahjong shRNA cells undergo apoptosis when they are cultured with normal cells in the presence of tetracycline.** MDCK pTR Mahjong shRNA cells were fluorescently labeled with CMFDA (green), mixed with normal MDCK cells at a ratio of 1∶10, and cultured in the presence of tetracycline. Time-lapse images were captured at 10 min intervals for 60 h, with fluorescent images captured every 30 min. (http://www.ucl.ac.uk/lmcb/images/Movie2.mov)
(4.36 MB MOV)

Video S3 **This video shows that Mahjong shRNA cells do not undergo apoptosis when they are cultured with normal cells in the absence of tetracycline.** MDCK pTR Mahjong shRNA cells were fluorescently labeled with CMFDA (green), mixed with normal MDCK cells at a ratio of 1∶10, and cultured in the absence of tetracycline. Time-lapse images were captured at 10 min intervals for 60 h, with fluorescent images captured every 30 min. (http://www.ucl.ac.uk/lmcb/images/Movie3.mov)
(5.56 MB MOV)

## Abbreviations

aPKC: atypical protein kinase C
DAPI: 4′, 6-diamidino-2-phenylindole dihydrochloride
Dlg: Discs large
FLP: flipase
EthD-1: ethidium homodimer-1
FRT: flipase recombination target
HEK cells: human embryonic kidney cells
HIV-1: human immunodeficiency virus type 1
JNK: c-Jun N-terminal kinase
Lgl: lethal giant larvae
LisH: Lis1 homology motif
MARCM: mosaic analysis with a repressible cell marker
MDCK: Madin-Darby canine kidney
mLgl: mammalian Lgl
Puc: Puckered
Vpr: viral protein R
VprBP: viral protein R-binding protein
