# Supplementary figures and images for: Involvement of Lgl and Mahjong/VprBP in Cell Competition
Source: PLoS Biol. 2010 Jul 13;8(7):e1000422. doi: 10.1371/journal.pbio.1000422 (PMC2903597; doi:10.1371/journal.pbio.1000422)

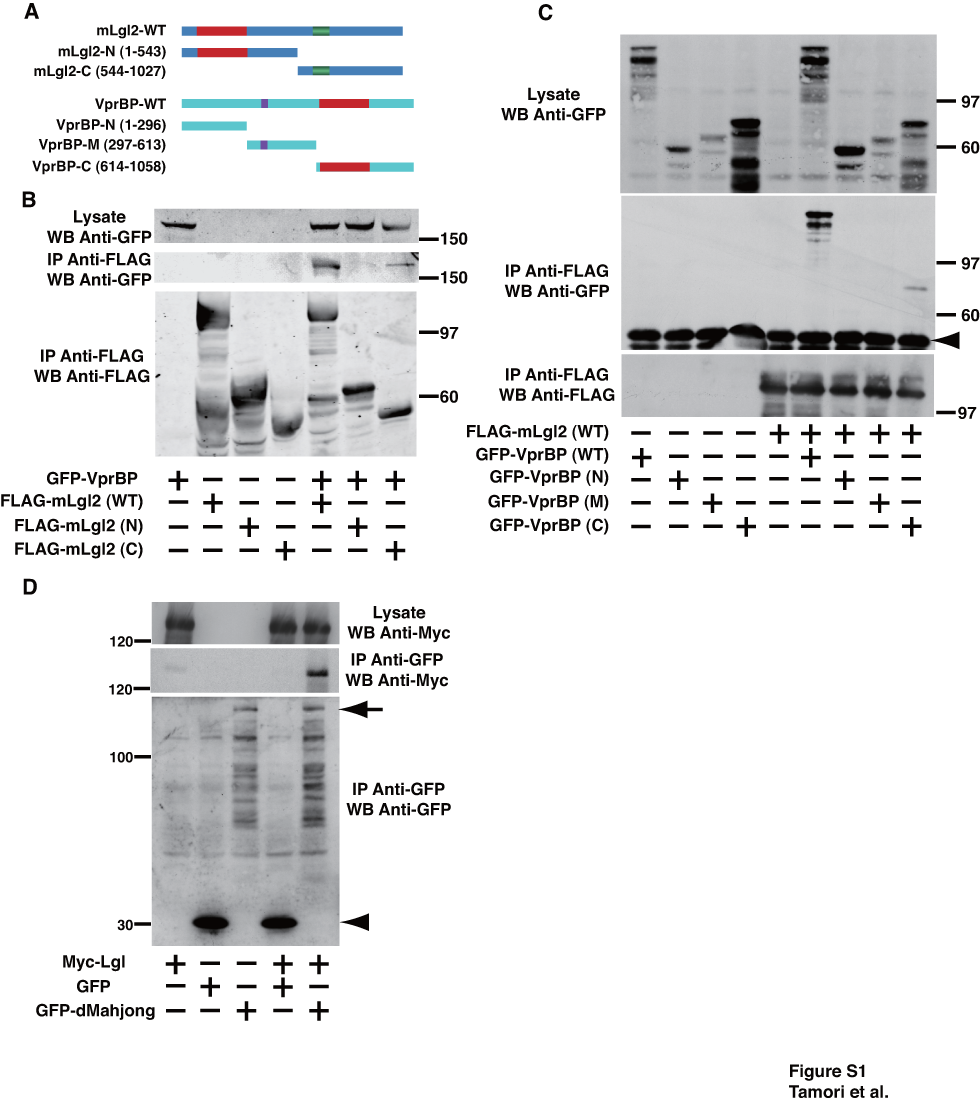

Supplement: Figure S1 — (A) A schematic illustrating the mLgl2 and VprBP constructs used (red, WD repeats; green, phosphorylation sites; purple, Lis1 homology motif domain). (B) Interaction of VprBP with the C-terminus of mLgl2. The full-length (FLAG-mLgl2-WT), N-terminus (FLAG-mLgl2-N), or C-terminus (FLAG-mLgl2-C) of mLgl2 was coexpressed with GFP-VprBP in human embryonic kidney (HEK) 293 cells. Immunoprecipitation was performed with anti-FLAG antibody, followed by Western blotting with anti-FLAG and anti-GFP antibodies. (C) Interaction of mLgl2 with the C-terminus of VprBP. The full-length (GFP-VprBP-WT), N-terminus (GFP-VprBP-N), middle part (GFP-VprBP-M), or C-terminus (GFP-VprBP-C) of VprBP was coexpressed with FLAG-mLgl2-WT in HEK293 cells. Immunoprecipitation was performed with anti-FLAG antibody, followed by Western blotting with anti-FLAG and anti-GFP antibodies. The arrowhead indicates the position of IgG heavy chains. (B and C) In human, two isoforms of VprBP are produced from the VprBP gene by alternative splicing. The shorter form consists of 1,058 amino acids and the longer form of 1,507 amino acids. The difference in length is due to an insertion of 449 amino acids after residue 225 in the long form. In these experiments, we used GFP-tagged shorter VprBP isoform. (D) Interaction of Lgl with Drosophila Mahjong protein. Myc-tagged Lgl was coexpressed with GFP or GFP-Mahjong in S2R+ cells, and immunoprecipitation was performed with anti-GFP antibody, followed by Western blotting with anti-Myc and anti-GFP antibodies. Mouse and rabbit anti-GFP antibodies were used for immunoprecipitation and Western blotting, respectively. The arrow and arrowhead indicate the positions of GFP-Mahjong and GFP, respectively. (0.45 MB TIF) [file pbio.1000422.s001.tif]

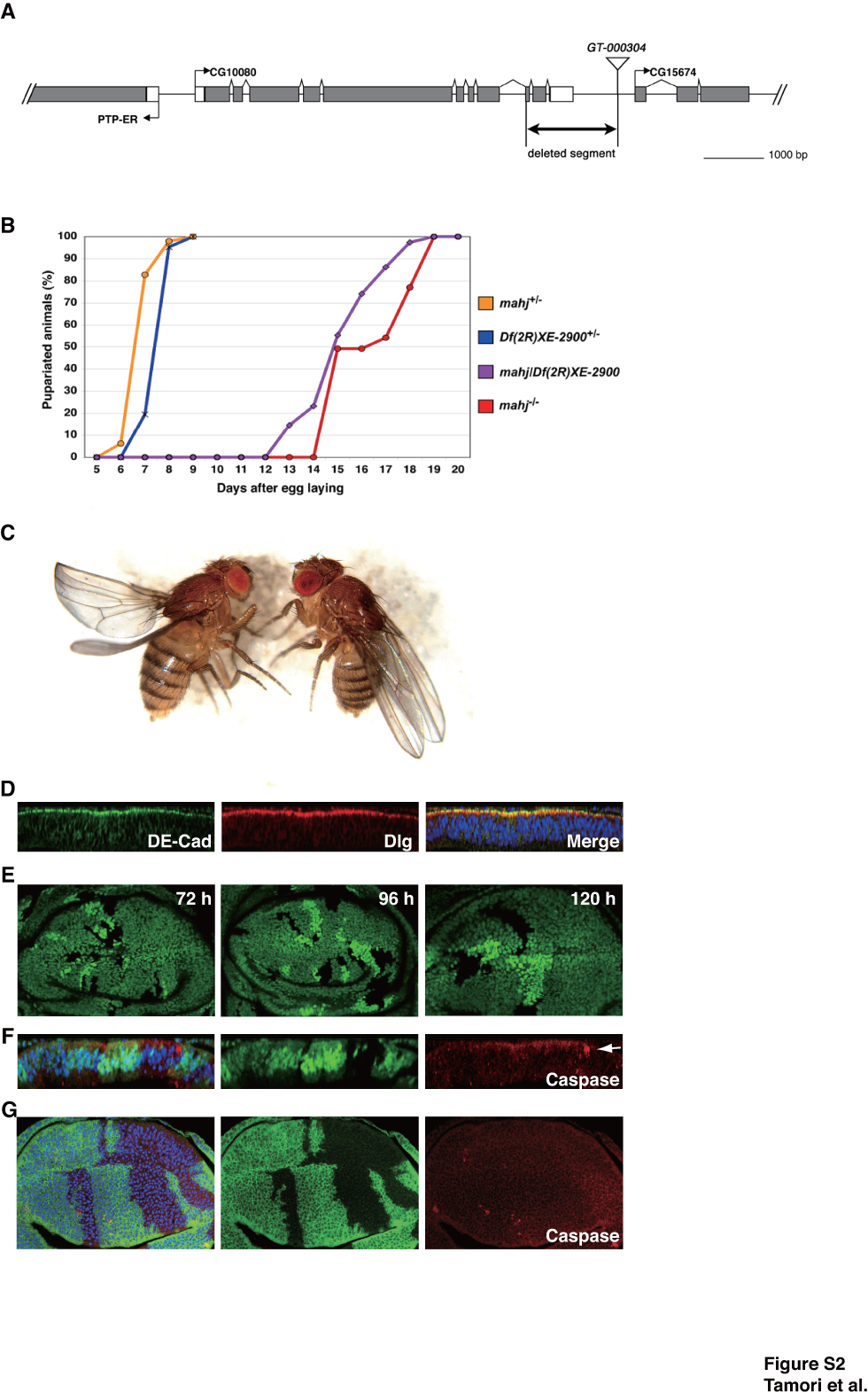

Supplement: Figure S2 — (A) Schematic representation of the genome region containing the mahjong (CG10080) locus. Predicted genes are indicated as boxes. The P-element insertion site of GT-000304 and the extent of the mahj1 deletion are indicated. (B) A line graph showing the growth defect of the homozygous mahj1 mutant larvae and the trans-heterozygous mutant larvae with Df(2R)XE-2900. Each value represents the percentage of pupariated animals on the indicated days after egg laying relative to the final number of pupae. Df(2R)XE-2900: a mutant of chromosomal deficiency in which the entire coding region of mahj is deleted. (C) Comparison of the heterozygous mahj1 control female (left) with the homozygous mahj1 mutant female rescued by expression of UAS-Mahjong under the control of actin-GAL4 (right). (D) Transverse sections of a wing disc of homozygous mahj1 larva that were immunostained with anti-DE-Cadherin and anti-Dlg antibodies. (E) Wing discs with wild-type (lacking GFP) and GFP-expressing wild-type clones at 72 h, 96 h, and 120 h (left to right) after clone induction. (F) Transverse sections of a wing disc with mahj −/− (lacking GFP) and wild-type clones (expressing GFP strongly) at 96 h after clone induction. The arrow indicates an apoptotic mahj −/− cell remaining within the epithelial monolayer. (G) A wing disc where mahj −/− clones are surrounded by Minute/+ heterozygous cells (expressing β-gal, green) at 144 h after clone induction. (D, F, and G) Nuclei were stained with DAPI (blue). (F and G) Apoptotic cells were labeled with anti-cleaved Caspase-3 antibody (red). (1.06 MB TIF) [file pbio.1000422.s002.tif]

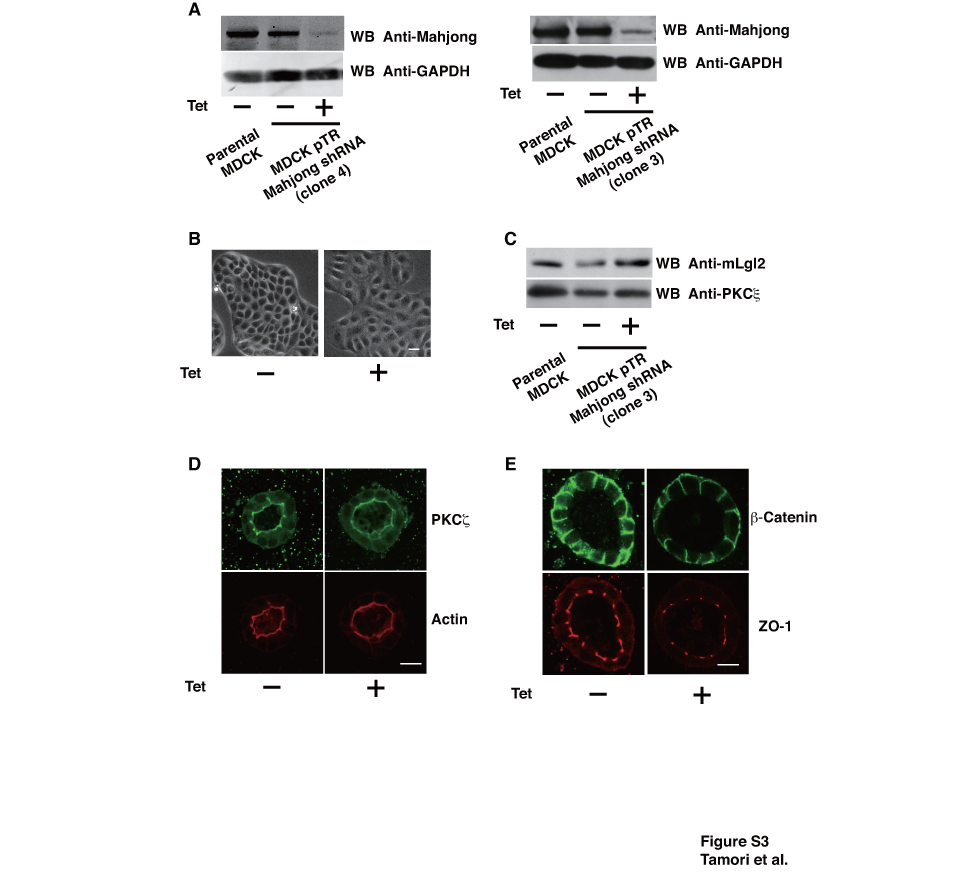

Supplement: Figure S3 — Characterization of the effect of Mahjong knockdown in MDCK epithelial cells. (A) Establishment of MDCK cell lines that stably express Mahjong shRNA in a tetracycline-inducible manner. Parental MDCK or MDCK pTR Mahjong shRNA cells (clone 3 or 4) were cultured with or without tetracycline for 48 h, and cell lysates were analyzed by Western blotting with anti-Mahjong or anti-GAPDH antibody. Note that comparable phenotypes were observed in cell polarity and cell competition with clones 3 and 4. (B) Effect of Mahjong knockdown on morphology in MDCK cells. MDCK pTR Mahjong shRNA cells were cultured with or without tetracycline for 48 h and were analyzed by phase-contrast microscopy. (C) Knockdown of Mahjong does not affect the expression of mLgl2 or PKCζ. Parental MDCK or MDCK pTR Mahjong shRNA cells were cultured with or without tetracycline for 48 h, and cell lysates were analyzed by Western blotting with anti-mLgl2 or anti-PKCζ antibody. (D and E) Immunofluorescence analyses of cell polarity markers in MDCK Mahjong shRNA cell cysts. MDCK pTR Mahjong shRNA cells were seeded in collagen gels and incubated with or without tetracycline for 11 d. Immunostaining was performed with anti-PKCζ antibody and TRITC-labeled phalloidin (D) or with anti-β-catenin and anti-ZO-1 antibodies (E). (B, D, and E) Scale bars: 10 µm. (0.30 MB TIF) [file pbio.1000422.s003.tif]

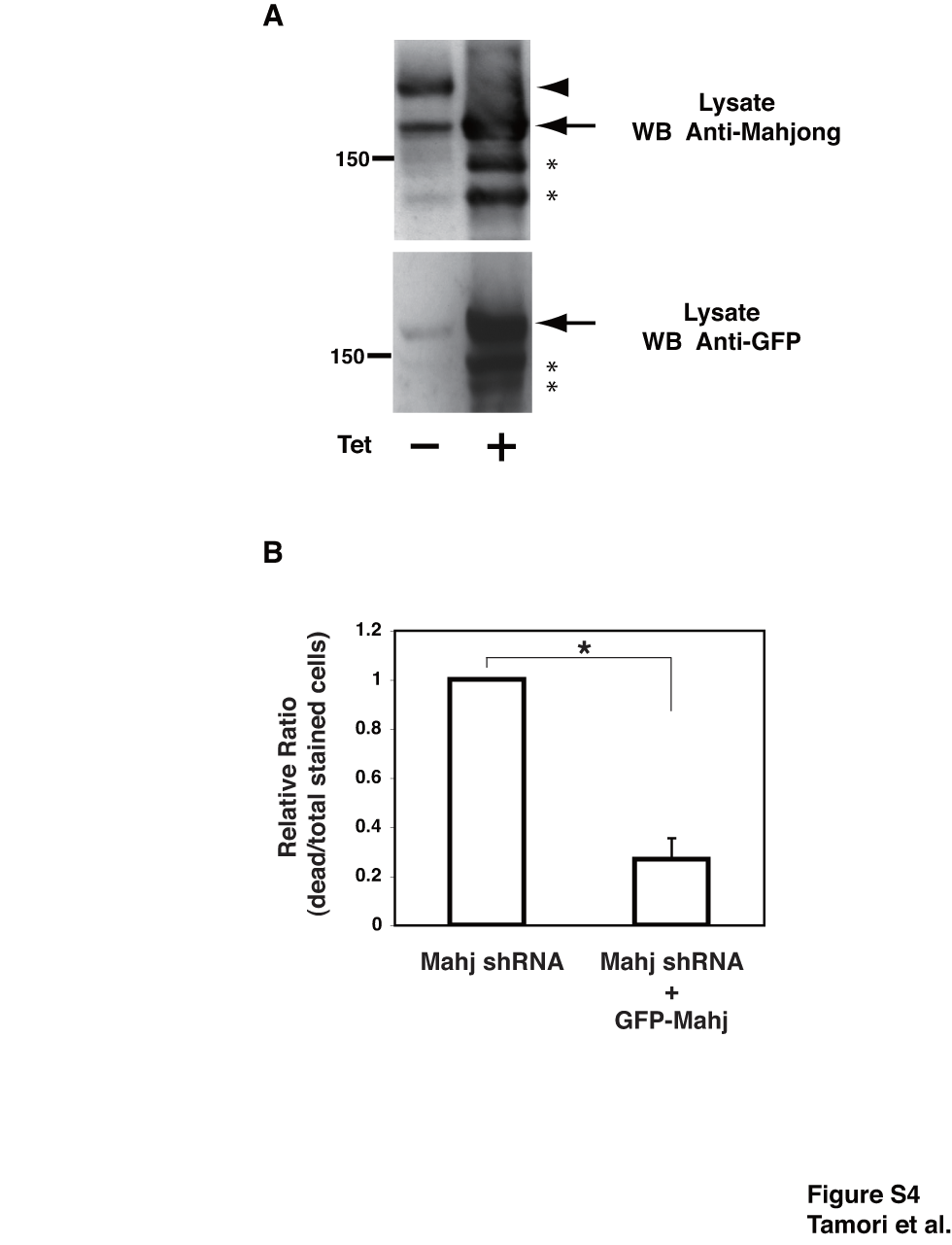

Supplement: Figure S4 — Overexpression of Mahjong alleviates the cell competition phenotype in MDCK Mahjong shRNA cells. (A) Establishment of MDCK pTR Mahjong shRNA+GFP-Mahjong cells that stably express Mahjong shRNA and GFP-tagged human Mahjong in a tetracycline-inducible manner. Because of mismatch of a base pair, expression of Mahjong shRNA does not knock down exogenously expressed human Mahjong. MDCK pTR Mahjong shRNA+GFP-Mahjong cells were cultured with or without tetracycline for 48 h, and cell lysates were analyzed by Western blotting with anti-Mahjong or anti-GFP antibody. Arrowhead, arrows, and asterisks indicate the positions of endogenous Mahjong protein, exogenously expressed GFP-Mahjong protein, and its degradation products, respectively. Note that MDCK cells predominantly express the longer Mahjong isoform and that the GFP-tagged shorter isoform of human Mahjong is exogenously expressed. This result therefore suggests that the shorter Mahjong isoform can substitute for the longer isoform in cell competition. (B) Effect of overexpression of exogenous Mahjong on cell death and apical extrusion of MDCK pTR Mahjong shRNA cells that are surrounded by normal MDCK cells. Fluorescently labeled MDCK pTR Mahjong shRNA cells or MDCK pTR Mahjong shRNA+GFP-Mahjong cells were mixed with normal MDCK cells, and cultured in the presence of tetracycline for 60 h. Frequency of cell death that occurred in fluorescently labeled cells was analyzed for 50–120 cells in each experimental condition. Values are expressed as a ratio relative to MDCK pTR Mahjong shRNA cells, and the results represent the means±SD of three independent experiments. *p<0.005. Note that all and only dead cells were apically extruded. (0.17 MB TIF) [file pbio.1000422.s004.tif]

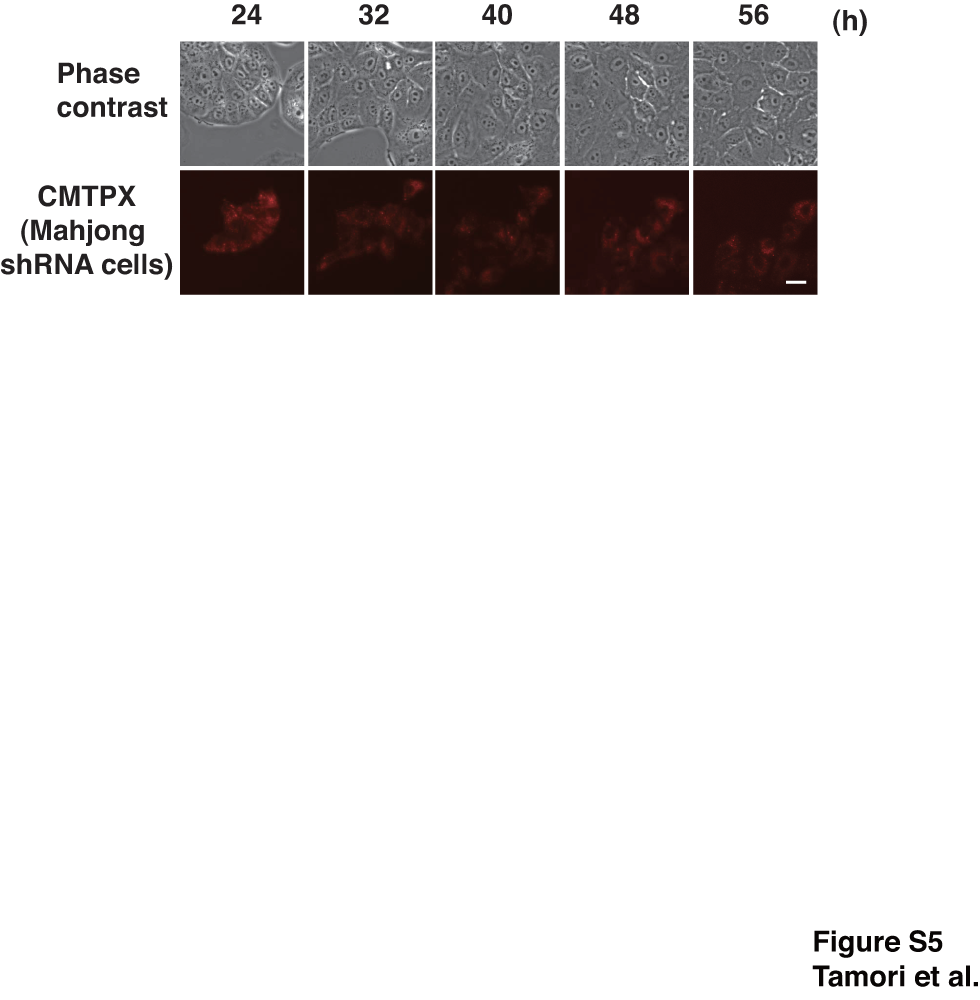

Supplement: Figure S5 — MDCK pTR Mahjong shRNA cells were fluorescently labeled with CMTPX (red), mixed with MDCK pTR Mahjong shRNA cells at a ratio of 1∶10, and cultured in the presence of tetracycline for the indicated times. Images were extracted from a representative time-lapse analysis. CMTPX was used in this experiment because MDCK pTR Mahjong shRNA cells express a low level of GFP. Scale bars: 30 µm. (0.29 MB TIF) [file pbio.1000422.s005.tif]

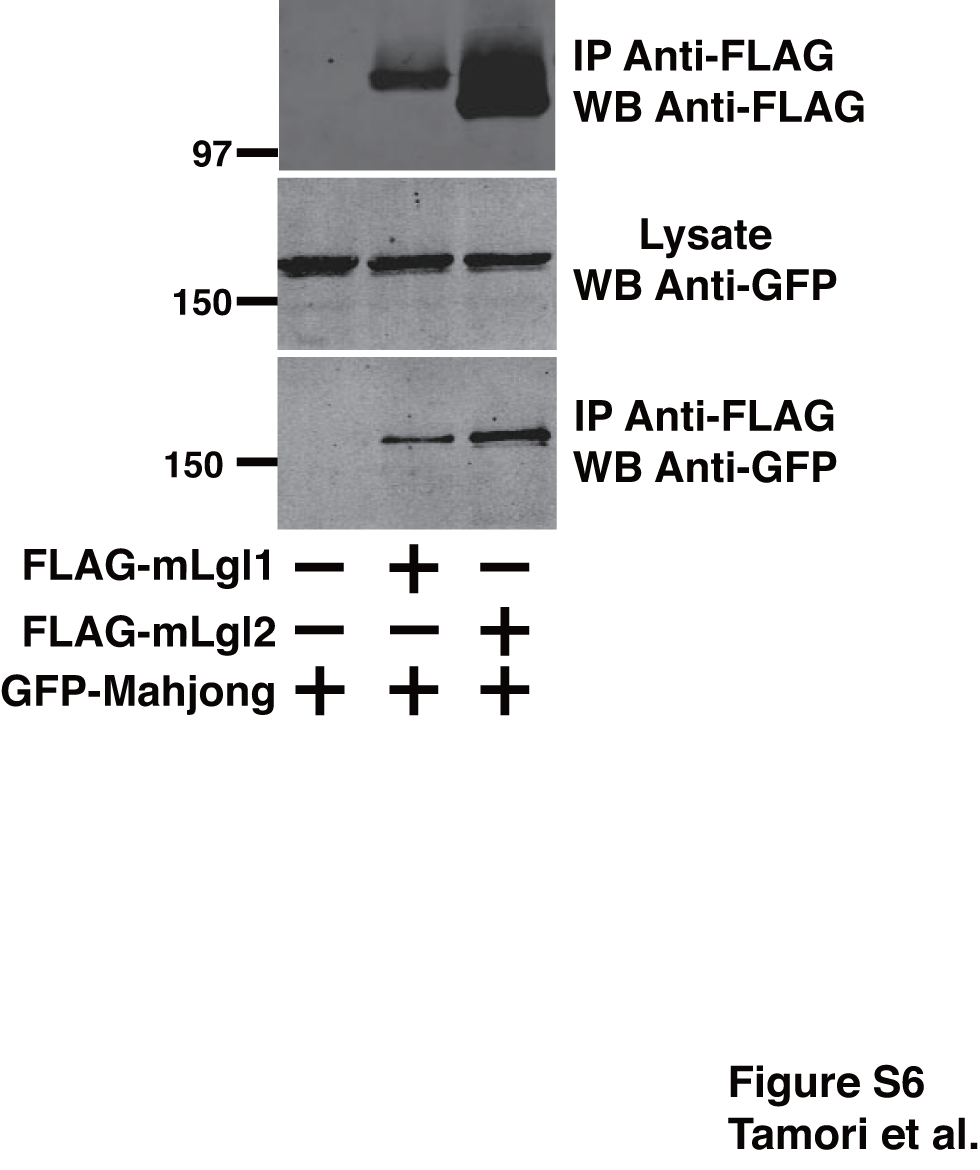

Supplement: Figure S6 — Mahjong interacts with both mLgl1 and mLgl2. GFP-Mahjong was coexpressed with FLAG-mLgl1 or FLAG-mLgl2 in HEK293 cells, and immunoprecipitation was performed with anti-FLAG antibody, followed by Western blotting with anti-FLAG and anti-GFP antibodies. (0.25 MB TIF) [file pbio.1000422.s006.tif]

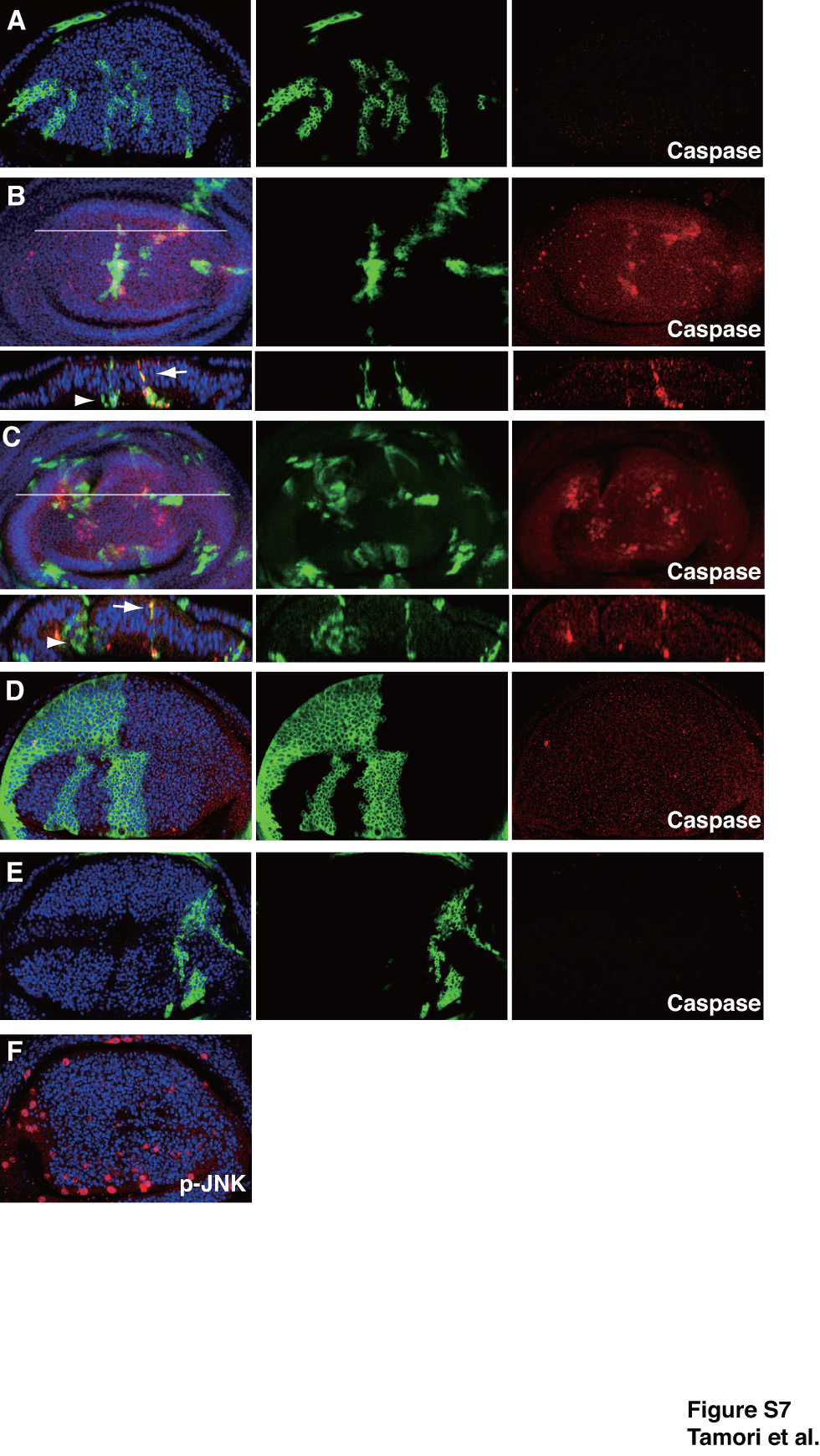

Supplement: Figure S7 — Analyses of mahj −/− or lgl −/− clones with the MARCM system expressing UAS-GFP. (A–C) Wild-type (A), mahj −/− (B), or lgl −/− (C) MARCM clones at 96 h after clone induction. (B and C) Arrows indicate apoptotic mahj −/− (B) or lgl −/−cells (C) remaining within the epithelial layer. Arrowheads indicate basally extruded mahj −/− (B) or lgl −/−cells (C) that were not stained with anti-active Caspase 3 antibody. (D) mahj −/− MARCM clones overexpressing Mahj 120 h after clone induction. (E) lgl −/− MARCM clones overexpressing Lgl at 120 h after clone induction. (F) A wild-type wing disc immunostained with anti-p-JNK antibody. Apoptotic cells were labeled with anti-cleaved Caspase-3 antibody (red), except in (F) where anti-p-JNK antibody (red) was used for immunostaining. (B and C) A Z-stack projection of 40 confocal images of a wing disc. (A–F) Nuclei were stained with DAPI (blue). (2.01 MB TIF) [file pbio.1000422.s007.tif]

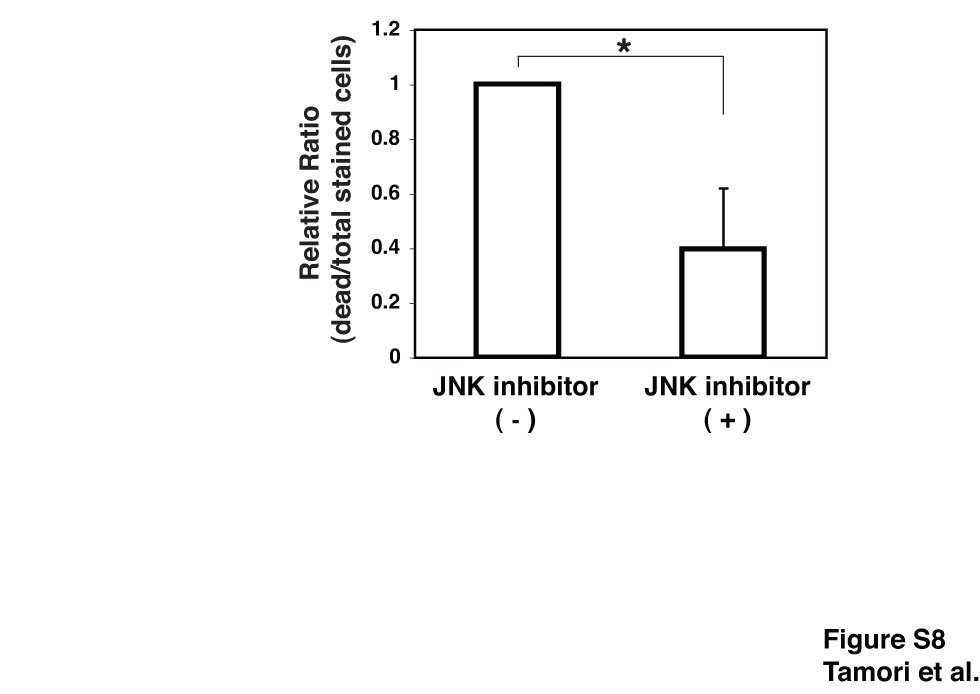

Supplement: Figure S8 — Effect of JNK inhibitor on Mahjong knockdown-mediated cell competition in MDCK cells. Fluorescently labeled MDCK pTR Mahjong shRNA cells were mixed with normal MDCK cells, and cultured with tetracycline in the absence or presence of JNK inhibitor (SP600125) for 60 h. Frequency of cell death that occurred in labeled MDCK pTR Mahjong shRNA cells was analyzed for 30–60 cells in each experimental condition. Values are expressed as a ratio relative to those in the absence of JNK inhibitor, and the results represent the means±SD of three independent experiments. *p<0.05. Note that all and only dead cells were apically extruded. (0.08 MB TIF) [file pbio.1000422.s008.tif]

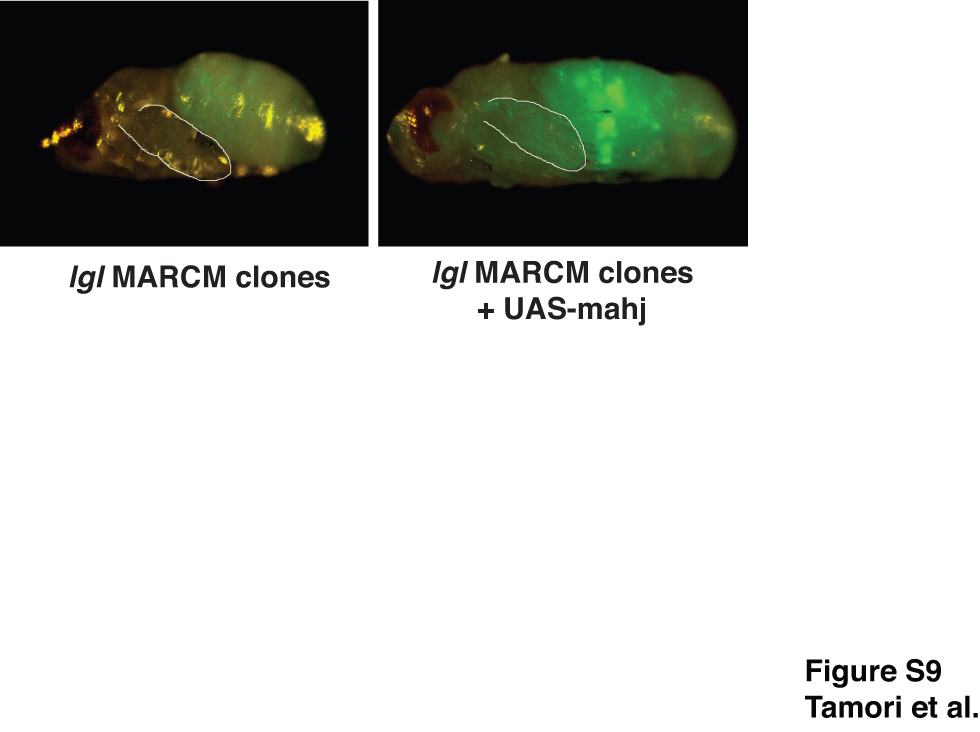

Supplement: Figure S9 — Mahj overexpression allows survival of lgl −/− clones to adulthood. lgl −/− MARCM clones expressing UAS-GFP without (left) or with (right) UAS-mahj in the pharate adult. Note that GFP-positive lgl −/− clones are observed within the wing (outlined with a white line) in the right panel, but not in the left panel. (0.20 MB TIF) [file pbio.1000422.s009.tif]

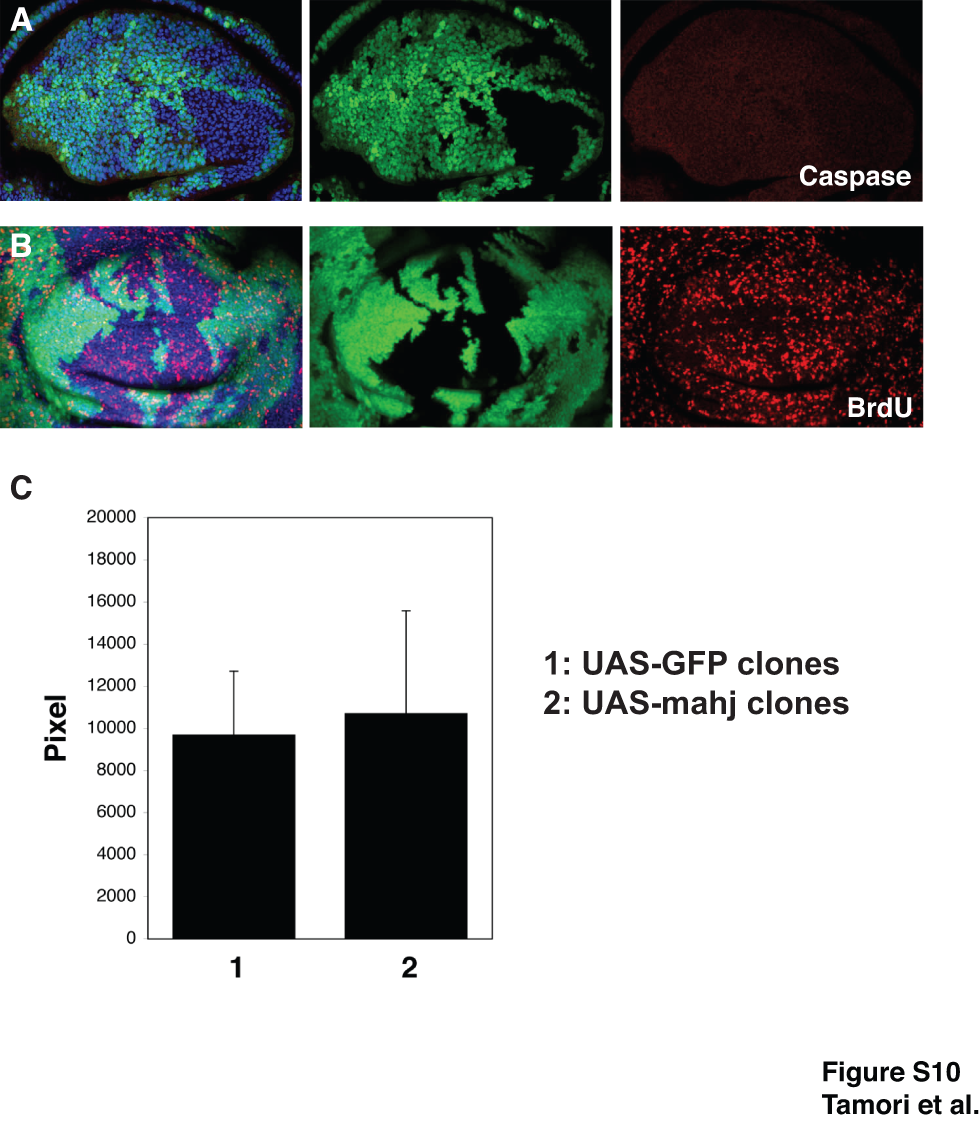

Supplement: Figure S10 — Overexpression of Mahj in wild-type cells does not induce apoptosis nor affect cell growth. (A and B) Wing discs with Mahj overexpression clones (green) at 96 h after clone induction. The Mahj overexpression clones were induced by hsFLP-Flip-out Gal4. (A) Apoptotic cells were labeled with anti-cleaved Caspase-3 antibody (red). (B) BrdU incorporation was detected by anti-BrdU antibody (red). (A and B) Nuclei were stained with DAPI (blue). (C) Quantification of the size of clones overexpressing UAS-mahjong or UAS-GFP in wild-type wing discs at 48 h after heat-shock. The results represent means±SD (n = 30 discs for each experimental condition). (0.91 MB TIF) [file pbio.1000422.s010.tif]

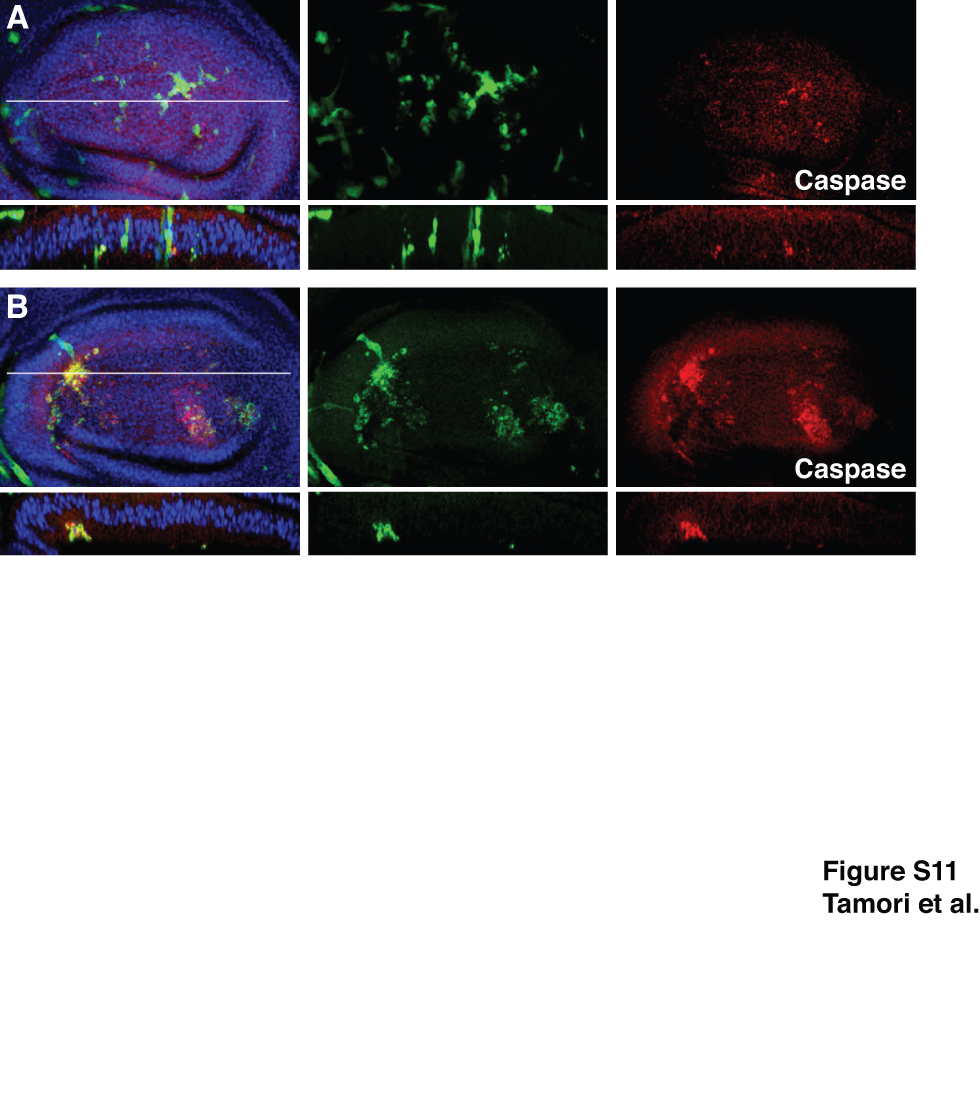

Supplement: Figure S11 — Inhibitory effect of Mahjong overexpression on apoptosis is specific to lgl −/− clones. The mosaic analysis with a repressible cell marker (MARCM) system was used to overexpress UAS constructs in either scrib −/− clones (A) or lgl −/− clones (B), and homozygous mutant clones are marked by the expression of GFP. (A) Overexpression of Mahj in scrib −/− clones at 72 h after clone induction. (B) Overexpression of dMyc in lgl −/− clones at 120 h after clone induction. Upper panels: A Z-stack projection of 40 confocal images of a wing disc. Lower panels: Transverse sections of the white line. Anti-cleaved Caspase-3 antibody was used for immunostaining (red). Nuclei were stained with DAPI (blue). (0.89 MB TIF) [file pbio.1000422.s011.tif]
